# Subtype-Specific Roles of Ellipsoid Body Ring Neurons in Sleep Regulation in *Drosophila*

**DOI:** 10.1523/JNEUROSCI.1350-22.2022

**Published:** 2023-02-01

**Authors:** Wei Yan, Hai Lin, Junwei Yu, Timothy D. Wiggin, Litao Wu, Zhiqiang Meng, Chang Liu, Leslie C. Griffith

**Affiliations:** ^1^Brain Cognition and Brain Disease Institute, Shenzhen Institute of Advanced Technology, Chinese Academy of Sciences, Shenzhen-Hong Kong Institute of Brain Science-Shenzhen Fundamental Research Institutions, Shenzhen, 518000, China; ^2^Central Research Institute, United Imaging Healthcare, Shanghai, 200032, China; ^3^Department of Biology, National Center for Behavioral Genomics and Volen Center for Complex Systems, Brandeis University, Waltham, Massachusetts 02453; ^4^CAS Key Laboratory of Brain Connectome and Manipulation, Shenzhen, 518000, China; ^5^Shenzhen Key Laboratory of Viral Vectors for Biomedicine, Shenzhen, 518000, China; ^6^Shenzhen Key Laboratory of Drug Addiction, Shenzhen, 518000, China

**Keywords:** central complex, *Drosophila melanogaster*, ellipsoid body, ring neurons, sleep, sleep structure

## Abstract

The ellipsoid body (EB) is a major structure of the central complex of the *Drosophila melanogaster* brain. Twenty-two subtypes of EB ring neurons have been identified based on anatomic and morphologic characteristics by light-level microscopy and EM connectomics. A few studies have associated ring neurons with the regulation of sleep homeostasis and structure. However, cell type-specific and population interactions in the regulation of sleep remain unclear. Using an unbiased thermogenetic screen of EB drivers using female flies, we found the following: (1) multiple ring neurons are involved in the modulation of amount of sleep and structure in a synergistic manner; (2) analysis of data for ΔP(doze)/ΔP(wake) using a mixed Gaussian model detected 5 clusters of GAL4 drivers which had similar effects on sleep pressure and/or depth: lines driving arousal contained R4m neurons, whereas lines that increased sleep pressure had R3m cells; (3) a GLM analysis correlating ring cell subtype and activity-dependent changes in sleep parameters across all lines identified several cell types significantly associated with specific sleep effects: R3p was daytime sleep-promoting, and R4m was nighttime wake-promoting; and (4) R3d cells present in 5HT7-GAL4 and in GAL4 lines, which exclusively affect sleep structure, were found to contribute to fragmentation of sleep during both day and night. Thus, multiple subtypes of ring neurons distinctively control sleep amount and/or structure. The unique highly interconnected structure of the EB suggests a local-network model worth future investigation; understanding EB subtype interactions may provide insight how sleep circuits in general are structured.

**SIGNIFICANCE STATEMENT** How multiple brain regions, with many cell types, can coherently regulate sleep remains unclear, but identification of cell type-specific roles can generate opportunities for understanding the principles of integration and cooperation. The ellipsoid body (EB) of the fly brain exhibits a high level of connectivity and functional heterogeneity yet is able to tune multiple behaviors in real-time, including sleep. Leveraging the powerful genetic tools available in *Drosophila* and recent progress in the characterization of the morphology and connectivity of EB ring neurons, we identify several EB subtypes specifically associated with distinct aspects of sleep. Our findings will aid in revealing the rules of coding and integration in the brain.

## Introduction

Sleep plays critical roles in many physiological functions. Sleep regulation in the brain is a complex process modulated at the molecular, cellular, circuit, and network levels (John et al., 2016; Scammell et al., 2017; Bringmann, 2018; Herice and Sakata, 2019; D. Liu and Dan, 2019). Previous studies in *Drosophila melanogaster* have revealed multiple cell types and neural circuits that participate in the regulation of sleep amount, structure, and homeostasis.

The ellipsoid body (EB) contributes to regulation of multiple behaviors, including spatial orientation, navigation, arousal, and sleep (Bausenwein et al., 1994; Lebestky et al., 2009; Ofstad et al., 2011; Seelig and Jayaraman, 2015; Fisher et al., 2019; Kim et al., 2019; Kottler et al., 2019). As one of the central structures on the midline of the fly brain, the EB receives direct input from, and sends output to, many brain regions. This high level of connectivity positions the EB to be a center for integration of multiple information streams, including visual, motor, mechanosensory, and circadian input, allowing it to functionally tune complex behaviors (Franconville et al., 2018).

The organization within the EB also exhibits complexity. With recent progress on morphology and connectivity of the EB, 22 distinct subtypes of ring neurons have been identified (Hulse et al., 2021). Each subtype of ring neuron typically contains a dendritic arborization lateral to the EB, then projects a single axon into the concentric laminated structure within the EB neuropil. The projections from each subtype of ring neuron form distinct layers within the neuropil, terminating in different rings at specific depths along the anterior-posterior axis where they interconnect (Hanesch et al., 1989; Young and Armstrong, 2010; Lin et al., 2013). These connections, between neurons of the same type, provide each ring neuron's strongest inputs (Isaacman-Beck et al., 2020; Hulse et al., 2021) and suggest a structural basis for local communication and synergism for sleep regulation.

Despite the growing understanding of EB connectivity, specific roles for each subtype of ring neuron in sleep are limited. One subtype of R5 neuron (initially referred to as R2) has been shown to drive a persistent sleep on secession of thermoactivation, suggesting a role in sleep drive and homeostasis (Donlea et al., 2014; S. Liu et al., 2016; Pimentel et al., 2016). Another study showed that single R5 neurons get synchronized by circadian input and the power of slow-wave oscillations in R5 neurons has been associated with increased sleep drive (Raccuglia et al., 2019). 5HT7-GAL4^+^ EB neurons, which consist of several subtypes including R3d, R3p, and R4d and are modulated by serotonergic signaling, can regulate sleep architecture (C. Liu et al., 2019). Despite these important findings, the scope of ring neuron involvement in the regulation of sleep is not clear.

In the present study, we take an unbiased approach, screening 34 drivers that label different combinations of subtypes of ring neurons by thermoactivation using the warmth-sensitive cation channel dTrpA1 (Hamada et al., 2008). Most drivers label multiple ring neurons, and activation of many drivers resulted in significant changes in sleep amount and/or sleep structure. The complexity of the tools and phenotypes necessitated developing computational approaches for assessing the importance of each subtype. Using P(wake) and P(doze) analysis with a mixed Gaussian model, five clusters of drivers were found to regulate sleep depth and pressure during the day and/or at night, respectively. Furthermore, a GLM analysis based on the GAL4 expression pattern and the sleep behavior on 24 h activation suggests several types of ring neuron contribute to sleep regulation consistent with and extending the findings from the Gaussian model. Finally, using genetic suppression of intersected population strategy, we identified a subpopulation of neurons which is sufficient to fragment sleep during both day and night. Although how the ring neurons cooperate to coherently modulate sleep is not yet clear, the identification of roles for specific cell types provides an important piece of the puzzle.

## Materials and Methods

### Animals

Unless specified, flies were reared on standard cornmeal food (each 1 L H_2_O: 70 g cornmeal, 50 g sucrose, 10 g soybean powder, 20 g yeast powder, 6 g agar, and 3 g methyl 4-hydroxybenzoate) at 23°C with 60% relative humidity and under a regimen of 12 h light/12 h dark. Flies were allowed to freely mate after eclosion, and mated females aged 2-5 d were used for all experiments. GAL4 lines: R12B01 (RRID:BDSC_48487), R15B07 (RRID:BDSC_48678), R28D01 (RRID:BDSC_47342), R28E01 (RRID:BDSC_49457), R38B06 (RRID:BDSC_49986), R38G08 (RRID:BDSC_50020), R38H02 (RRID:BDSC_47352), R41A08 (RRID:BDSC_50108), R41F05-GAL4 (RRID:BDSC_50133), R47F07 (RRID:BDSC_50320), R48B10 (RRID:BDSC_50352), R49E12 (RRID:BDSC_38693), R53F11 (RRID:BDSC_50443), R53G11 (RRID:BDSC_69747), R54B05 (RRID:BDSC_69148), R56C09 (RRID:BDSC_39145), R64H04 (RRID:BDSC_39323), R70B04 (RRID:BDSC_39513), R70B05 (RRID:BDSC_47721), R73A06 (RRID:BDSC_39805), R73B05 (RRID:BDSC_48312), R81F01 (RRID:BDSC_40120), R84H09 (RRID:BDSC_47803), Aph^c507^ (RRID:BDSC_30840), C232 (RRID:BDSC_30828), and R44D11-LexA (RRID:BDSC_41264), UAS-dTrpA1 (RRID:BDSC_26263), UAS-mCD8::GFP (RRID:BDSC_5136), UAS-mCD8::RFP, LexAop2-mCD8::GFP (RRID:BDSC_32229), and LexAop-Gal80 (RRID:BDSC_32213) were ordered from the Bloomington Drosophila Stock Center. GAL4 lines: VT012446, VT026841, VT042577, VT042759, VT045108, VT057257, VT038828, VT040539, and VT059775 were ordered from Vienna Drosophila Resource Center originally, but unfortunately not available anymore. 5HT7-GAL4 was provided by Charles Nicols' laboratory. Feb170-GAL4 was generated by Günter Korge's laboratory (Siegmund and Korge, 2001). The WT line *w^CS^* was crossed with GAL4 and UAS parental lines as genetic controls. Experimental groups were from the F1 generation of crosses of GAL4 lines to UAS-dTrpA1.

### Experimental design for sleep assays and calculation of sleep changes

F1 generation of flies were all maintained on standard food at 23°C. Two- to 5-day-old mated F1 female flies were individually placed into a 65 mm × 5 mm glass tube containing food (2% agar and 5% sucrose). After loading to the DAM2 system (Drosophila Activity Monitor) (Trikinetics; https://www.trikinetics.com/) at 21°C in 12 h:12 h light/dark cycles, flies were entrained for 2-3 d. Then 1 d baseline sleep, 1 d neural activation sleep, as well as 1 d recovery sleep were recorded at 21°C, 30°C, and 21°C, respectively. Total sleep, the number of sleep episodes, and maximum episode length were analyzed for light and dark periods (LP and DP) separately, using MATLAB (RRID:SCR_001622) program (SCAMP2019v2) scripts.

To overview the effects on activation of GAL4^+^ neurons, all genotypes were arranged in a descending order according to the changes of total sleep during the LP. Sleep changes were calculated by subtracting baseline day sleep of each genotype from its activation day. Since using TRPA1 to activate neurons requires an elevation of ambient temperature (above 25°C), and temperature has been shown to effect sleep (Parisky et al., 2016; Jin et al., 2021; Alpert et al., 2022), it is critical to compare with control groups that have undergone the same temperature shift. With genetic control groups and a subtraction to the baseline day, the temperature effect can be removed and sleep changes because of activation of the neurons can be quantified. For genotypes with significant changes in sleep and/or sleep structure, 3 days' sleep profiles of sleep time in 30 min were plotted. Sleep changes of the recovery day were also calculated. The significant difference was marked when the experimental group is different compared with both genetic controls.

### Immunohistochemistry

Brains of adult flies were dissected in 10 mm ice-cold PBS and fixed for 20 min in PBS with 4% PFA at room temperature. Brains were then washed 3 times for 5 min each in PBT (PBS with 0.5% Triton X-100). For GFP and RFP immunostaining, brains were incubated with primary antibodies (1:200, chicken anti-GFP, Abcam, catalog #ab13970, RRID:AB_300798; 1:200, mouse anti-GFP, Roche, catalog #11814460001, RRID:AB_390913; 1:1000, rabbit anti-GFP, Invitrogen, catalog #A-11122, RRID:AB_221569; 1:200, rabbit anti-DsRed, Takara, catalog #632496, RRID:AB_10013483) in 10% NGS in PBT at 4°C for two nights. After 3 times washes for 5 min each with PBT at room temperature, brains were incubated with secondary antibody at 4°C overnight. Second antibodies (488 goat anti-mouse, Invitrogen, catalog #A-11001, RRID:AB_2534069; 488 goat anti-chicken, Invitrogen, catalog #A-11039, RRID:AB_142924; 488 goat anti-rabbit, Invitrogen, catalog #A-11008, RRID:AB_143165; 568 goat anti-rabbit, Fisher Scientific, catalog #A-11011, RRID:AB_143157) were all used in a ratio of 1:200. Samples were then washed 3 times for 5 min each in PBT at room temperature, and mounted on microscope slide in Vectashield mounting medium (Vector Laboratories catalog #H-1000, RRID:AB_2336789). Finally, samples were imaged with Leica TCS SP5/LSM900 confocal microscope (RRID:SCR_002140) and analyzed using the open source of FIJI (ImageJ) software (RRID:SCR_002285).

### Probability analysis

The probability of transitioning from a sleep to an awake state (P(wake)), and from a wake state to a sleep state (P(doze)) was used power law distributions analysis as previously described (Wiggin et al., 2020). P(wake) and P(doze) were calculated identically, with calculation of 1 min bin of inactivity and activity reversed. The MATLAB scripts for analysis of P(wake)/P(doze) can be accessed in GitHub at https://github.com/Griffith-Lab/Fly_Sleep_Probability.

### Mixed Gaussian model clustering

To figure out different effects of EB drivers on both sleep pressure and depth, we divided all significant subtypes of EB ring neurons into groups with similar distributions of δ P(Wake) and δ P(Doze), using mixed Gaussian model clustering. The clustering analysis was conducted using the scripts of fitgmdist and cluster in MATLAB. Given the small sample size of neuron subtypes (14 and 13 for daytime and nighttime, respectively), the number of cluster *k* was set to 3, 4, or 5 for both daytime and nighttime. We calculated the silhouette coefficients for each *k* value using the script of silhouette in MATLAB and chose the final *k* value whose silhouette coefficient was the closest to one (Lecompte et al., 1986). The size of ellipse for each cluster was decided by the corresponding σ values of its Gaussian mixture distribution.

### GLM

To evaluate the effect of a specific anatomic subtype of ring neurons on sleep, the GLM (Generalized linear models) was used to estimate the weights and the corresponding statistical significance of all subtypes for each sleep parameter. The GLM analysis was conducted using the script of glmfit in MATLAB (The MathWorks) to predict each sleep parameter under the combination of all subtypes of neurons. The input variable was defined as 1 or 0 for each subtype of ring neurons (R1, R2, R3d, R3m, R3a, R3p, R3w, R4m, R4d, R5, and R6) when labeled or not labeled by each driver, respectively. And the corresponding output variable was the mean change rate of each sleep parameter of the same driver on the activation to its baseline level (output variable value = (activation – baseline)/baseline). We chose the default parameters for the script of glmfit. According to the weight calculation for each subtype (see Table 6), a positive value represents positive relationship, and a negative value represents negative relationship between the subtype and the sleep parameter, respectively, when the corresponding *p* value < 0.05.

### Statistical analysis

Power analysis was conducted using the script of sampsizepwr in MATLAB (The MathWorks) to calculate the power for the sample size in this study. The power analysis was based on the sleep parameters in drivers with significant differences from both control groups presented in the main figures. We selected the mean and SD of control groups under the null hypothesis, and the mean value of experimental groups under the alternative hypothesis during the calculation of power values. Based on current sample size, >80% of the powers of significances of sleep parameters were >0.9 (see Tables 2 and 7).

Data were performed using GraphPad Prism 8 (RRID:SCR_002798). Group means were compared using one-way ANOVA followed by Bonferroni's multiple comparison test when data were normally distributed, or Kruskal–Wallis test followed by Dunn's multiple comparison test was used when data failed passing normality test (see Tables 1, 3, 4, 8, and 9). All experiments were performed at least 2 replicates, and data presented in the figures were chosen from one representative replicate. To uniform the data presentation, all figures were prepared as mean ± SEM. To visualize all groups in the same figure clearer, error bars were not shown.

## Results

### Thermoactivation of ring neurons changes sleep amount

**Figure 1.**
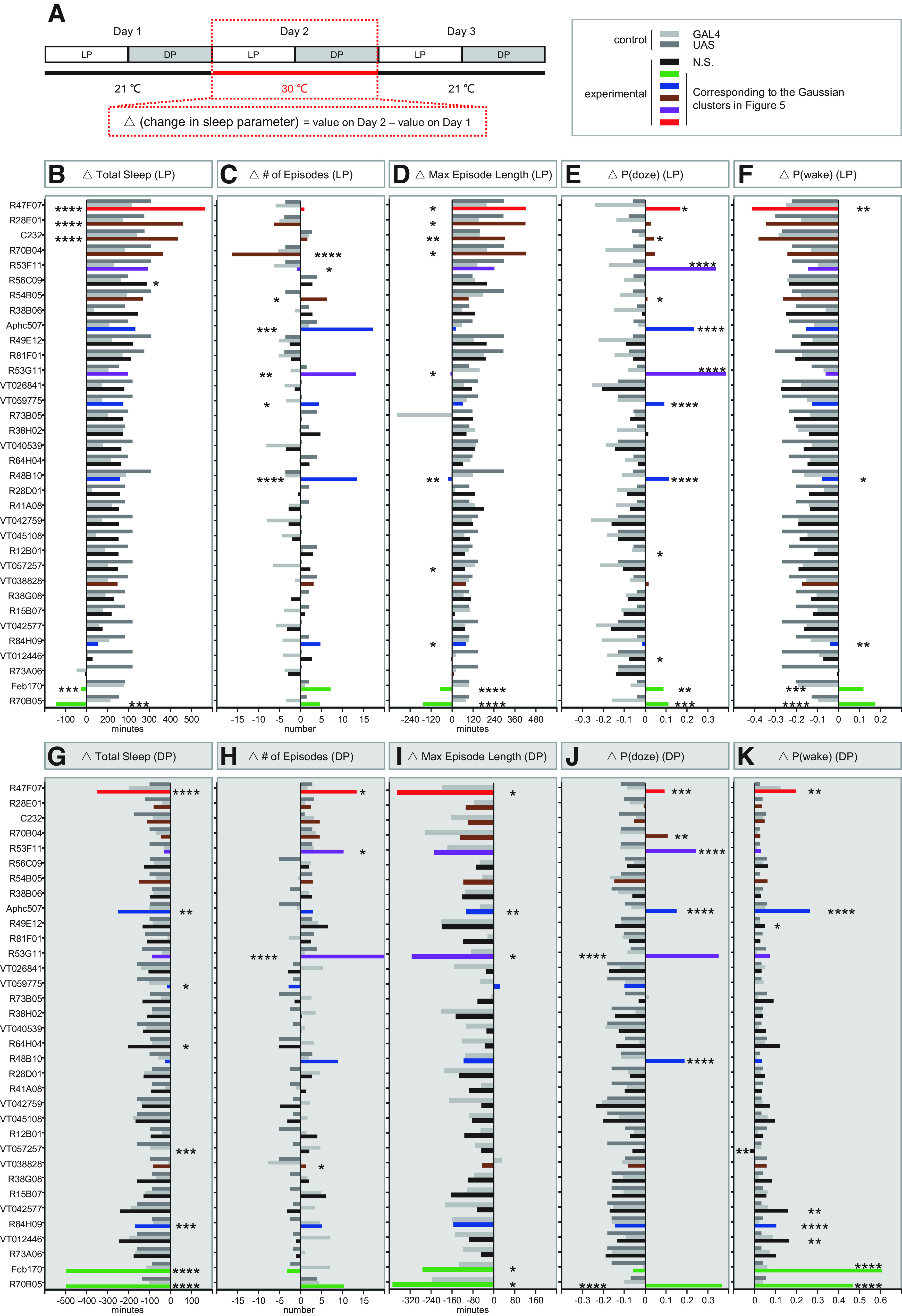
Sleep changes with activation of subtypes of ring neurons. ***A***, Design of the experiments and calculation of sleep parameters on the activation day (red dashed box). ***B***, ***G***, Changes in sleep amount during day (LP) and night (DP). ***C***, ***H***, Changes in number of sleep episodes during daytime and night. ***D***, ***I***, Changes in maximum sleep episodes during daytime and night. ***E***, ***J***, Changes in P(doze) during daytime and night. ***F***, ***K***, Changes in P(wake) during daytime and night. Colored and black bars represent the experimental groups. Color codes are consistent through all of the figures and are based on the daytime cluster analysis in Figure 5. Gray and dark gray bars represent GAL4 control and UAS control, respectively. One-way ANOVA analysis and Dunn's multiple comparisons test were used. Significance, only when the experimental group is significantly different from both GAL4 and UAS controls: *p < 0.05. **p < 0.01. ***p < 0.001. ****p < 0.0001. Data are mean.

**Figure 2.**
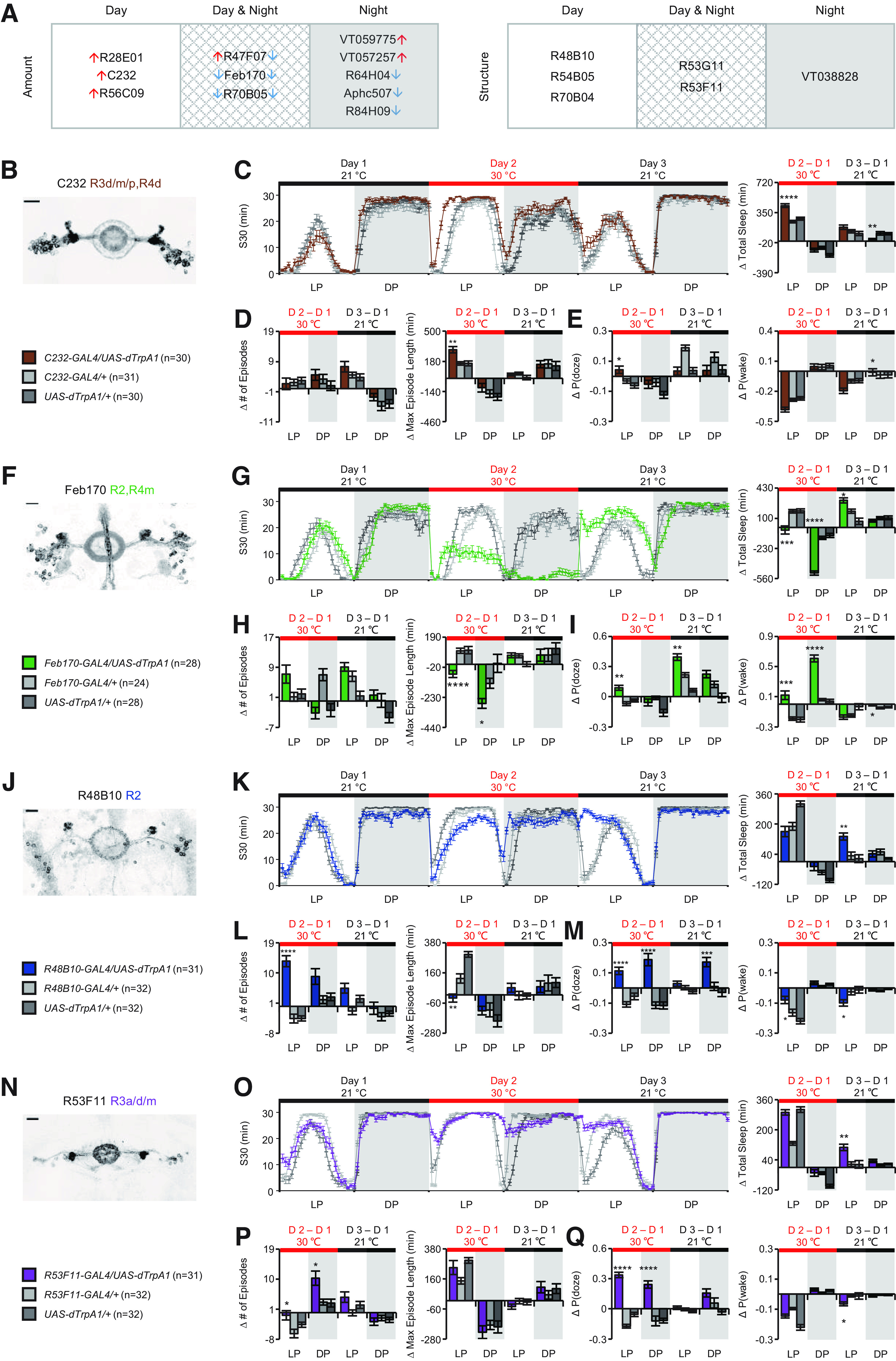
Complex effects on sleep homeostasis with thermoactivation of ring neurons. ***A***, Summary of the drivers exhibited significant changes in total amount of sleep and sleep structure, respectively. Arrows on the left and right represent changes during the day and night, respectively. Up arrows represent increased total amount of sleep. Down arrows represent decreased total amount of sleep. Clusters represent the phenotypes observed day only, night only, or both day and night. Expression patterns of c232-GAL4 (***B***), Feb170-GAL4 (***F***), R48B10-GAL4 (***J***), and R53F11-GAL4 (***N***). Sleep profiles with quantification of changes of sleep parameters of each driver: total sleep (***C***,***G***,***K***,***O***), the number of episodes and maximum episode length (***D***,***H***,***L***,***P***), and P(doze) and P(wake) (***E***,***I***,***M***,***Q***). Scale bar, 20 μm. *p < 0.05. **p < 0.01. ***p < 0.001. ****p < 0.0001.

**Figure 3.**
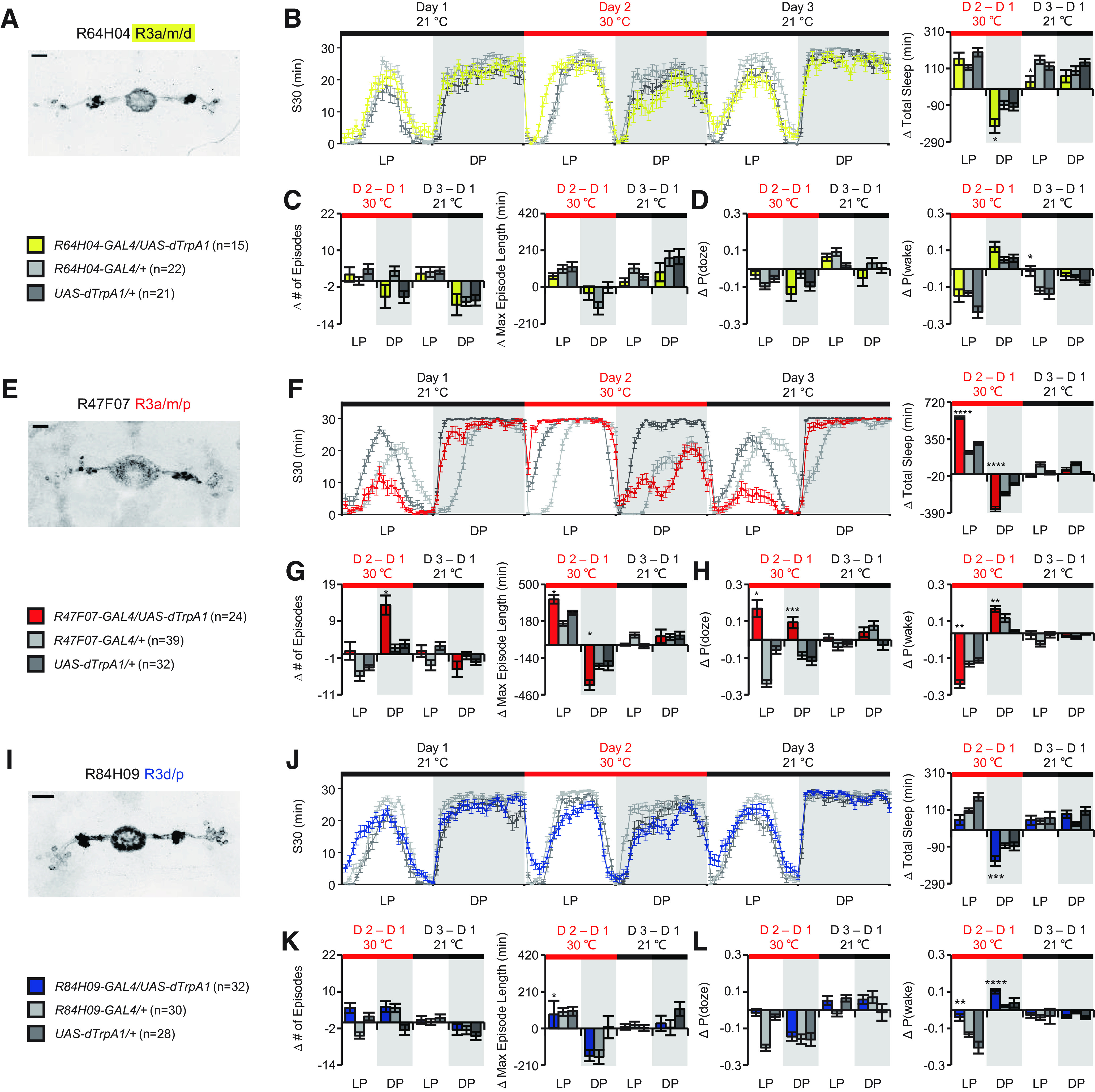
Complex effects on sleep homeostasis with thermoactivation of ring neurons. ***A–D***, Decreased nighttime sleep often fails to induce rebound sleep on cessation of thermoactivation of ring neurons. ***A***, Expression pattern of R64H04-GAL4 which labels R3a, R3m, and R3d neurons. ***B***, Sleep profile and quantification of total sleep before, during, and after activation. Activation of R64H04-GAL4^+^ neurons reduced total sleep at night, and a persisting reduced sleep on cessation of activation. ***C***, No significant change was observed in the number of episodes and maximum episode length. ***D***, No change of P(doze) was observed, and significantly higher change in P(wake) than controls was found on cessation of activation. ***E–L***, Drivers involved in the regulation of sleep amount and/or structure do not exhibit homeostatic rebound on cessation of thermoactivation. Expression pattern, sleep profile, quantification of sleep amount, sleep structure, and sleep drive/arousal threshold of each driver were presented. ***E–H***, R47F07-GAL4. ***I–L***, R84H09-GAL4. *p < 0.05. **p < 0.01. ***p < 0.001. ****p < 0.0001. Data are mean ± SEM. Scale bar: 20 μm.

To investigate the roles of ring neuron types, we collected 34 GAL4 drivers that label different populations of ring neurons and used them to drive the thermogenetic tool dTrpA1, allowing the use of elevated temperature to drive neuronal firing (Hamada et al., 2008). Animals were placed in DAM2 system tubes and entrained at 21°C in a 12 h:12 h light/dark cycle. Sleep was then recorded for 3 d: 1 d of baseline sleep at 21°C, 1 d of neural activation sleep at 30°C, then 1 d of recovery sleep at 21°C (Fig. 1*A*). Changes in sleep parameters for each genotype on the activation and recovery days were calculated by subtracting the baseline day value (Fig. 1*A*). Changes were only considered significant when the experimental group was different from both genetic controls. Changes in total daytime sleep of the 34 drivers on the activation day are arranged in descending order (Fig. 1*B*), and changes of total nighttime sleep (Fig. 1*G*) as well as changes in the number of episodes (Fig. 1*C*,*H*), maximum episode duration (Fig. 1*D*,*I*), P(doze) (Fig. 1*E*,*J*), and P(wake) (Fig. 1*F*,*K*) are displayed in the same order as the daytime sleep data to allow assessment of all parameter changes for each genotype. The color-coding of the histogram bars corresponds to the Gaussian clusters shown in Figure 5 and is also used to identify lines in Figures 2, 3, 6, and 7 as part of particular clusters.

Activation of GAL4^+^ neurons produced many different patterns of change in the amount of sleep. During the daytime, a significant increase in total sleep was found when R47F07-GAL4^+^, R28E01-GAL4^+^, C232-GAL4^+^, and R56C09-GAL4^+^ neurons were activated (Fig. 1*B*; Table 1). Since change in total sleep is often associated with change in sleep structure (C. Liu et al., 2019; Wiggin et al., 2020), we also evaluated the number of sleep episodes, episode length, and the behavioral transition probabilities, P(doze) and P(wake) (Wiggin et al., 2020) to further understand the changes in sleep drive and arousal threshold. The increased sleep observed in the above three drivers was accompanied by a significant increase in maximum episode length but no change in the number of episodes compared with their genetic controls (Fig. 1*C*,*D*). These flies had increased P(doze) and decreased P(wake), suggesting that these neurons possibly contribute to increased sleep pressure and sleep depth (Fig. 1*E*,*F*).

**Table 1.** Statistical analysis of the activation day for 34 drivers used in Figure 1*^a^*

△ Total sleep	LP (Fig. 1*B*)										DP (Fig. 1*G*)					
	Nonparametric/parametric test				*Post hoc* comparisons						Nonparametric/parametric test			*Post hoc* comparisons		
Driver	Test	DFn, DFd	*F*	*p*		n1	n2	Mean difference	*p*		Test	*F*	*p*	Mean difference	*p*	
R47F07	ANOVA	2,92	118	<0.0001	1 vs 2	24	39	350.6	<0.0001	****	ANOVA	46.59	<0.0001	−152.8	<0.0001	****
					1 vs 3	24	32	257.9	<0.0001	****				−249.2	<0.0001	****
R28E01	K-W	3,95	54.91	<0.0001	1 vs 2	32	31	51.12	<0.0001	****	K-W	10.74	0.0046	−15.22	0.0569	NS
					1 vs 3	32	32	30.41	<0.0001	****				7.094	0.6067	NS
C232	K-W	3,91	31.94	<0.0001	1 vs 2	30	31	36.11	<0.0001	****	K-W	10.32	0.0057	−3.126	>0.9999	NS
					1 vs 3	30	30	29.27	<0.0001	****				17.13	0.024	*
R70B04	K-W	3,94	43.46	<0.0001	1 vs 2	30	32	43.7	<0.0001	****	K-W	8.177	0.0168	6.665	0.6727	NS
					1 vs 3	30	32	11.9	0.2195	NS				19.43	0.0101	*
R53F11	K-W	3,95	51.75	<0.0001	1 vs 2	31	32	40.65	<0.0001	****	K-W	23.45	<0.0001	1.31	>0.9999	NS
					1 vs 3	31	32	−4.4	>0.9999	NS				29.62	<0.0001	****
R56C09	ANOVA	2,51	5.698	0.0058	1 vs 2	22	11	125.5	0.0077	**	ANOVA	0.579	0.5642	−44.95	0.5146	NS
					1 vs 3	22	21	89.39	0.0232	*				−27.91	0.6812	NS
R54B05	K-W	3,89	22.79	<0.0001	1 vs 2	26	31	19.65	0.0085	**	K-W	12.29	0.0021	−24.07	0.0009	***
					1 vs 3	26	32	−11.12	0.2059	NS				−12.41	0.1378	NS
R38B06	K-W	3,86	34.64	<0.0001	1 vs 2	29	29	37.19	<0.0001	****	K-W	2.427	0.2972	−8.966	0.3431	NS
					1 vs 3	29	28	9.566	0.2964	NS				−8.772	0.3697	NS
Aphc507	K-W	3,77	13.32	0.0013	1 vs 2	28	28	21.04	0.0009	***	K-W	21.59	<0.0001	−24.18	0.0001	***
					1 vs 3	28	21	4.952	0.8863	NS				−25.18	0.0002	***
R49E12	ANOVA	2,93	26.93	<0.0001	1 vs 2	32	32	99.88	0.0003	***	ANOVA	3.407	0.0373	−47.66	0.0236	*
					1 vs 3	32	32	−86.66	0.0019	**				−33.16	0.1413	NS
R81F01	K-W	3,96	16.46	0.0003	1 vs 2	32	32	10.72	0.2475	NS	K-W	18.51	<0.0001	−25.95	0.0004	***
					1 vs 3	32	32	−17.28	0.0262	*				−0.01563	>0.9999	NS
R53G11	K-W	3,96	11.82	0.0027	1 vs 2	32	32	23.38	0.0016	**	K-W	19.26	<0.0001	−20.84	0.0055	**
					1 vs 3	32	32	7.188	0.604	NS				8.938	0.3987	NS
VT026841	ANOVA	2122	30.52	<0.0001	1 vs 2	31	31	105.9	<0.0001	****	ANOVA	3.524	0.0325	37.39	0.1971	NS
					1 vs 3	31	63	−39.68	0.0634	NS				54.29	0.0167	*
VT059775	ANOVA	2114	20.89	<0.0001	1 vs 2	28	26	103.2	0.0003	***	ANOVA	16.31	<0.0001	85.44	0.01	*
					1 vs 3	28	63	−43.83	0.0888	NS				142.6	<0.0001	****
R73B05	ANOVA	2,51	3.395	0.0413	1 vs 2	17	16	72.13	0.1285	NS	ANOVA	2.099	0.133	−87.99	0.0842	NS
					1 vs 3	17	21	−23.49	0.7478	NS				−35.22	0.5896	NS
R38H02	K-W	3,86	0.235	0.889	1 vs 2	27	31	0.6565	>0.9999	NS	K-W	1.185	0.5529	−5.4	0.8227	NS
					1 vs 3	27	28	−2.376	>0.9999	NS				−6.99	0.5986	NS
VT040539	ANOVA	2121	30.93	<0.0001	1 vs 2	29	32	87.69	0.0001	***	ANOVA	2.882	0.0599	−15.65	0.7011	NS
					1 vs 3	29	63	−53.56	0.0093	**				28.5	0.2546	NS
R64H04	ANOVA	2,55	3.678	0.0317	1 vs 2	15	22	48.58	0.2599	NS	ANOVA	4.723	0.0128	−113.7	0.0111	*
					1 vs 3	15	21	−35.03	0.4794	NS				−104.1	0.0222	*
R48B10	K-W	3,95	24.6	<0.0001	1 vs 2	31	32	−3.269	>0.9999	NS	K-W	19.05	<0.0001	15.12	0.0591	NS
					1 vs 3	31	32	−31.21	<0.0001	****				30.32	<0.0001	****
R28D01	K-W	3,90	32.67	<0.0001	1 vs 2	32	30	31.6	<0.0001	****	K-W	4.767	0.0922	0.05104	>0.9999	NS
					1 vs 3	32	28	−3.527	>0.9999	NS				−12.96	0.1104	NS
R41A08	K-W	3,88	6.072	0.048	1 vs 2	32	28	8.607	0.3858	NS	K-W	4.948	0.0842	−13.51	0.0819	NS
					1 vs 3	32	28	−8.214	0.428	NS				−1.172	>0.9999	NS
VT042759	ANOVA	2118	21.26	<0.0001	1 vs 2	27	31	80.45	0.0069	**	ANOVA	0.487	0.6156	2.115	0.996	NS
					1 vs 3	27	63	−65.97	0.0117	*				21.63	0.6054	NS
VT045108	ANOVA	2120	38.59	<0.0001	1 vs 2	28	32	107.2	<0.0001	****	ANOVA	0.528	0.591	12.84	0.805	NS
					1 vs 3	28	63	−67.04	0.0031	**				−8.04	0.8938	NS
R12B01	K-W	3,78	12.95	0.0015	1 vs 2	32	25	15.31	0.0227	*	K-W	6.563	0.0376	−14.36	0.0352	*
					1 vs 3	32	21	−8.075	0.4089	NS				−0.7254	>0.9999	NS
VT057257	ANOVA	2123	19.46	<0.0001	1 vs 2	31	32	47.47	0.0708	NS	ANOVA	23.47	<0.0001	98.3	0.0004	***
					1 vs 3	31	63	−70.94	0.001	**				161.2	<0.0001	****
VT038828	K-W	3,62	5.49	0.0643	1 vs 2	26	15	5.356	0.7197	NS	K-W	4.588	0.1008	−9.454	0.2121	NS
					1 vs 3	26	21	−8.482	0.2182	NS				3.346	>0.9999	NS
R38G08	K-W	3,83	10.76	0.0046	1 vs 2	26	29	8.57	0.376	NS	K-W	6.406	0.0406	−15.45	0.0353	*
					1 vs 3	26	28	−12.3	0.1221	NS				−13.08	0.0928	NS
R15B07	K-W	3,85	14.76	0.0006	1 vs 2	29	28	11.53	0.1559	NS	K-W	5.134	0.0768	−3.488	>0.9999	NS
					1 vs 3	29	28	−13.78	0.0702	NS				−14.26	0.0585	NS
VT042577	K-W	3120	58.2	<0.0001	1 vs 2	27	31	9.205	0.6295	NS	K-W	12.25	0.0022	−19.2	0.0719	NS
					1 vs 3	27	62	−43.13	<0.0001	****				−28.07	0.0009	***
R84H09	K-W	3,90	18.2	0.0001	1 vs 2	32	30	−12.04	0.1397	NS	K-W	17.35	0.0002	−23.64	0.0007	***
					1 vs 3	32	28	−28.79	<0.0001	****				−24.29	0.0007	***
VT012446	ANOVA	2117	75.08	<0.0001	1 vs 2	28	29	30.76	0.341	NS	ANOVA	7.622	0.0008	−48.21	0.1101	NS
					1 vs 3	28	63	−191	<0.0001	****				−85.36	0.0004	***
R73A06	K-W	3120	75.21	<0.0001	1 vs 2	25	32	8.963	0.6687	NS	K-W	1.036	0.5958	−3.546	>0.9999	NS
					1 vs 3	25	63	−49.77	<0.0001	****				−7.989	0.6624	NS
Feb170	K-W	3,80	18.24	0.0001	1 vs 2	28	24	−22.63	0.0009	***	K-W	51.75	<0.0001	−35.42	<0.0001	****
					1 vs 3	28	28	−23.77	0.0003	***				−41.73	<0.0001	****
R70B05	K-W	3,92	26.7	<0.0001	1 vs 2	28	32	−26.62	0.0002	***	K-W	53.05	<0.0001	−47.15	<0.0001	****
					1 vs 3	28	32	−34.32	<0.0001	****				−40.02	<0.0001	****

△ No. of episodes	LP (Fig. 1*C*)										DP (Fig. 1*H*)					
	Nonparametric/parametric test				*Post hoc* comparisons						Nonparametric/parametric test			*Post hoc* comparisons		
Driver	Test	DFn, DFd	*F*	*p*		n1	n2	Mean difference	*p*		Test	*F*	*p*	Mean difference	*p*	
R47F07	K-W	3,95	7.568	0.0227	1 vs 2	24	39	19.64	0.0119	*	K-W	18.4	0.0001	29.67	<0.0001	****
					1 vs 3	24	32	12.74	0.1729	NS				24.76	0.0017	**
R28E01	K-W	3,95	2.654	0.2653	1 vs 2	32	31	−4.62	>0.9999	NS	K-W	0.739	0.6911	1.878	>0.9999	NS
					1 vs 3	32	32	−11.16	0.2099	NS				−3.953	>0.9999	NS
C232	K-W	3,91	0.678	0.7125	1 vs 2	30	31	3.171	>0.9999	NS	K-W	2.079	0.3536	2.794	>0.9999	NS
					1 vs 3	30	30	−2.367	>0.9999	NS				9.55	0.3222	NS
R70B04	K-W	3,94	35.74	<0.0001	1 vs 2	30	32	−32.64	<0.0001	****	K-W	0.779	0.6776	3.006	>0.9999	NS
					1 vs 3	30	32	−38.64	<0.0001	****				6.1	0.7555	NS
R53F11	K-W	3,95	9.895	0.0071	1 vs 2	31	32	20.93	0.0051	**	K-W	9.558	0.0084	18.14	0.0178	*
					1 vs 3	31	32	15.93	0.0432	*				19.06	0.012	*
R56C09	K-W	3,54	2.258	0.3233	1 vs 2	22	11	5.568	0.6736	NS	K-W	6.358	0.0416	−4.023	0.9765	NS
					1 vs 3	22	21	−3.209	>0.9999	NS				9.295	0.1051	NS
R54B05	K-W	3,89	26.26	<0.0001	1 vs 2	26	31	18.94	0.0114	*	K-W	4.334	0.1145	10.62	0.2432	NS
					1 vs 3	26	32	34.87	<0.0001	****				−2.155	>0.9999	ns
R38B06	ANOVA	2,83	2.258	0.1109	1 vs 2	29	29	4.103	0.0839	NS	ANOVA	3.078	0.0514	0.7586	0.9196	NS
					1 vs 3	29	28	0.899	0.87	NS				5.187	0.0442	*
Aphc507	K-W	3,77	24.3	<0.0001	1 vs 2	28	28	27.54	<0.0001	****	K-W	7.434	0.0243	7.679	0.3972	NS
					1 vs 3	28	21	23.79	0.0005	***				17.59	0.0128	*
R49E12	K-W	3,96	1.664	0.4351	1 vs 2	32	32	8.453	0.4482	NS	K-W	6.715	0.0348	13.72	0.0972	NS
					1 vs 3	32	32	6.828	0.6522	NS				16.98	0.0292	*
R81F01	K-W	3,96	3.284	0.1936	1 vs 2	32	32	12.59	0.14	NS	K-W	9.191	0.0101	16.44	0.0362	*
					1 vs 3	32	32	6.484	0.7018	NS				−3.219	>0.9999	NS
R53G11	K-W	3,96	22.95	<0.0001	1 vs 2	32	32	32.3	<0.0001	****	K-W	44.77	<0.0001	44.39	<0.0001	****
					1 vs 3	32	32	23.25	0.0017	**				34.36	<0.0001	****
VT026841	K-W	3125	8.142	0.0171	1 vs 2	31	31	16.61	0.1409	NS	K-W	22.53	<0.0001	−36.65	0.0001	***
					1 vs 3	31	63	−5.966	0.9039	NS				−1.65	>0.9999	NS
VT059775	K-W	3117	12.24	0.0022	1 vs 2	28	26	31.59	0.0012	**	K-W	0.976	0.614	−8.577	0.7052	NS
					1 vs 3	28	63	19.7	0.0209	*				−2.032	>0.9999	NS
R73B05	K-W	3,54	2.625	0.2692	1 vs 2	17	16	0.5919	>0.9999	NS	K-W	5.997	0.0499	−6.77	0.4324	NS
					1 vs 3	17	21	−6.804	0.369	NS				5.99	0.4854	NS
R38H02	K-W	3,86	9.21	0.01	1 vs 2	27	31	19.9	0.0048	**	K-W	5.21	0.0739	−8.433	0.398	NS
					1 vs 3	27	28	10.16	0.2613	NS				6.322	0.6947	NS
VT040539	K-W	3124	24.96	<0.0001	1 vs 2	29	32	19.98	0.0599	NS	K-W	3.677	0.159	−3.832	>0.9999	NS
					1 vs 3	29	63	−18.53	0.0428	*				10.06	0.4235	NS
R64H04	ANOVA	2,55	1.367	0.2633	1 vs 2	15	22	2.088	0.6098	NS	ANOVA	4.35	0.0176	−8.248	0.0391	*
					1 vs 3	15	21	−1.724	0.713	NS				0.1238	0.999	NS
R48B10	K-W	3,95	46.85	<0.0001	1 vs 2	31	32	40.93	<0.0001	****	K-W	7.392	0.0248	17.16	0.0267	*
					1 vs 3	31	32	41.57	<0.0001	****				15.45	0.0517	NS
R28D01	K-W	3,90	3.352	0.1871	1 vs 2	32	30	−3.553	>0.9999	NS	K-W	9.549	0.0084	−5.297	0.8486	NS
					1 vs 3	32	28	−12.11	0.1453	NS				15.17	0.0493	*
R41A08	ANOVA	2,85	5.785	0.0044	1 vs 2	32	28	−0.1295	0.9951	NS	ANOVA	1.715	0.1861	2.393	0.3985	NS
					1 vs 3	32	28	−4.772	0.006	**				3.679	0.1318	NS
VT042759	K-W	3121	21.94	<0.0001	1 vs 2	27	31	20.81	0.0481	*	K-W	8.514	0.0142	−25.67	0.0108	*
					1 vs 3	27	63	−15.04	0.1239	NS				−8.14	0.6248	NS
VT045108	K-W	3123	8.938	0.0115	1 vs 2	28	32	9.393	0.6156	NS	K-W	3.369	0.1856	−15.18	0.1992	NS
					1 vs 3	28	63	−13.03	0.2138	NS				−2.889	>0.9999	NS
R12B01	K-W	3,78	3.412	0.1816	1 vs 2	32	25	9.155	0.2589	NS	K-W	12.76	0.0017	6.425	0.5756	NS
					1 vs 3	32	21	−2.077	>0.9999	NS				22.53	0.0008	***
VT057257	K-W	3126	22.64	<0.0001	1 vs 2	31	32	41.51	<0.0001	****	K-W	15.57	0.0004	−12.24	0.3661	NS
					1 vs 3	31	63	11.37	0.3106	NS				17.93	0.0502	NS
VT038828	ANOVA	2,59	1.74	0.1845	1 vs 2	26	15	4.382	0.2177	NS	ANOVA	5.011	0.0098	9.069	0.0096	**
					1 vs 3	26	21	−0.7418	0.9423	NS				6.46	0.0475	*
R38G08	ANOVA	2,80	1.988	0.1436	1 vs 2	26	29	−2.434	0.3897	NS	ANOVA	2.548	0.0846	0.3448	0.9817	NS
					1 vs 3	26	28	−4.121	0.0904	NS				4.429	0.0875	NS
R15B07	K-W	3,85	10.93	0.0042	1 vs 2	29	28	17.1	0.0176	*	K-W	14.44	0.0007	6.181	0.6876	NS
					1 vs 3	29	28	−3.076	>0.9999	NS				23.97	0.0005	***
VT042577	K-W	3120	19.98	<0.0001	1 vs 2	27	31	16.35	0.1474	NS	K-W	14.08	0.0009	−31.35	0.0012	**
					1 vs 3	27	62	−17.24	0.0625	NS				−7.047	0.7584	NS
R84H09	K-W	3,90	20.78	<0.0001	1 vs 2	32	30	29.04	<0.0001	****	K-W	11.58	0.0031	−1.535	>0.9999	NS
					1 vs 3	32	28	6.507	0.6706	NS				19.42	0.008	**
VT012446	K-W	3120	18.28	0.0001	1 vs 2	28	29	38.62	<0.0001	****	K-W	21.18	<0.0001	−29.3	0.0029	**
					1 vs 3	28	63	14.5	0.1319	NS				6.21	0.8629	NS
R73A06	K-W	3120	9.067	0.0107	1 vs 2	25	32	3.286	>0.9999	NS	K-W	4.374	0.1123	−8.631	0.7041	NS
					1 vs 3	25	63	−17.14	0.0736	NS				7.051	0.7813	NS
Feb170	ANOVA	2,77	3.437	0.0372	1 vs 2	28	24	6.054	0.0387	*	ANOVA	11.1	<0.0001	−10.18	0.0001	***
					1 vs 3	28	28	5.25	0.0666	NS				−0.75	0.9254	NS
R70B05	K-W	3,92	8.747	0.0126	1 vs 2	28	32	18.15	0.0171	*	K-W	2.965	0.2271	9.442	0.3428	NS
					1 vs 3	28	32	1.772	>0.9999	NS				11.15	0.2129	NS

△ Maximum episode length	LP (Fig. 1*D*)										DP (Fig. 1*I*)					
	Nonparametric/parametric test				*Post hoc* comparisons						Nonparametric/parametric test			*Post hoc* comparisons		
Driver	Test	DFn, DFd	*F*	*p*		n1	n2	Mean difference	*p*		Test	*F*	*p*	Mean difference	*p*	
R47F07	K-W	3,95	22.94	<0.0001	1 vs 2	24	39	33.99	<0.0001	****	K-W	8.847	0.012	−19.7	0.0117	*
					1 vs 3	24	32	17.46	0.038	*				−18.91	0.0221	*
R28E01	K-W	3,95	37.95	<0.0001	1 vs 2	32	31	42.57	<0.0001	****	K-W	4.146	0.1258	−1.289	>0.9999	NS
					1 vs 3	32	32	17.2	0.0251	*				11.5	0.1904	NS
C232	K-W	3,91	13.18	0.0014	1 vs 2	30	31	21.43	0.0031	**	K-W	2.027	0.3629	5.958	0.7569	NS
					1 vs 3	30	30	21.33	0.0035	**				9.617	0.317	NS
R70B04	K-W	3,94	21.76	<0.0001	1 vs 2	30	32	32.33	<0.0001	****	K-W	3.214	0.2005	12.38	0.1483	NS
					1 vs 3	30	32	16.28	0.0376	*				5.442	0.865	NS
R53F11	K-W	3,95	18.06	0.0001	1 vs 2	31	32	11.71	0.184	NS	K-W	0.858	0.6513	−6.345	0.7222	NS
					1 vs 3	31	32	−17.4	0.0245	*				−4.142	>0.9999	NS
R56C09	K-W	3,54	3.97	0.1374	1 vs 2	22	11	6.636	0.5066	NS	K-W	1.94	0.379	0.04545	>0.9999	NS
					1 vs 3	22	21	9.381	0.1013	NS				−6.102	0.4072	NS
R54B05	K-W	3,89	25.17	<0.0001	1 vs 2	26	31	−12.84	0.1231	NS	K-W	6.834	0.0328	−8.372	0.446	NS
					1 vs 3	26	32	−33.55	<0.0001	****				8.645	0.41	NS
R38B06	K-W	3,86	5.417	0.0667	1 vs 2	29	29	15.22	0.0405	*	K-W	2.062	0.3566	−1.224	>0.9999	NS
					1 vs 3	29	28	6.685	0.6245	NS				−8.794	0.3675	NS
Aphc507	K-W	3,77	8.111	0.0173	1 vs 2	28	28	−4	>0.9999	NS	K-W	15.37	0.0005	−17.68	0.0062	**
					1 vs 3	28	21	−17.85	0.0114	*				−23.57	0.0005	***
R49E12	K-W	3,96	21.38	<0.0001	1 vs 2	32	32	13.92	0.0912	NS	K-W	0.052	0.9745	−1.578	>0.9999	NS
					1 vs 3	32	32	−18.19	0.018	*				−0.6719	>0.9999	NS
R81F01	K-W	3,96	15.93	0.0003	1 vs 2	32	32	0.1719	>0.9999	NS	K-W	8.752	0.0126	−10.81	0.241	NS
					1 vs 3	32	32	−23.98	0.0011	**				9.781	0.3203	NS
R53G11	K-W	3,96	14.58	0.0007	1 vs 2	32	32	−26.19	0.0003	***	K-W	16.56	0.0003	−27.7	0.0001	***
					1 vs 3	32	32	−17.08	0.0284	*				−19.03	0.0126	*
VT026841	K-W	3125	11.13	0.0038	1 vs 2	31	31	13.68	0.2743	NS	K-W	12.01	0.0025	31.89	0.0011	**
					1 vs 3	31	63	−12.52	0.2302	NS				16.09	0.0859	NS
VT059775	K-W	3117	18.86	<0.0001	1 vs 2	28	26	−9.118	0.6472	NS	K-W	12.9	0.0016	18.48	0.0909	NS
					1 vs 3	28	63	−30.99	0.0001	***				27.66	0.0007	***
R73B05	K-W	3,54	2.125	0.3456	1 vs 2	17	16	7.397	0.3541	NS	K-W	3.092	0.2131	−6.57	0.4611	NS
					1 vs 3	17	21	1.171	>0.9999	NS				−8.835	0.1704	NS
R38H02	K-W	3,86	4.33	0.1147	1 vs 2	27	31	−13.12	0.092	NS	K-W	8.219	0.0164	6.246	0.684	NS
					1 vs 3	27	28	−3.622	>0.9999	NS				−12.2	0.1399	NS
VT040539	K-W	3124	1.149	0.5629	1 vs 2	29	32	−3.626	>0.9999	NS	K-W	3.704	0.1569	11.61	0.4152	NS
					1 vs 3	29	63	−8.339	0.6022	NS				15.48	0.1098	NS
R64H04	K-W	3,58	2.046	0.3595	1 vs 2	15	22	−7.117	0.4163	NS	K-W	5.632	0.0598	9.37	0.195	NS
					1 vs 3	15	21	−7.367	0.3938	NS				−2.267	>0.9999	NS
R48B10	K-W	3,95	43.17	<0.0001	1 vs 2	31	32	−21.71	0.0036	**	K-W	2.044	0.3598	1.821	>0.9999	NS
					1 vs 3	31	32	−45.61	<0.0001	****				9.336	0.358	NS
R28D01	K-W	3,90	8.714	0.0128	1 vs 2	32	30	19.28	0.0074	**	K-W	7.384	0.0249	7.048	0.5768	NS
					1 vs 3	32	28	6.165	0.7236	NS				−11.47	0.1796	NS
R41A08	K-W	3,88	7.201	0.0273	1 vs 2	32	28	10.37	0.2333	NS	K-W	1.163	0.5592	−3.431	>0.9999	NS
					1 vs 3	32	28	17.57	0.0157	*				−7.127	0.562	NS
VT042759	K-W	3121	1.694	0.4288	1 vs 2	27	31	3.568	>0.9999	NS	K-W	2.454	0.2932	14.21	0.2475	NS
					1 vs 3	27	63	−6.024	0.9105	NS				5.741	0.9534	NS
VT045108	K-W	3123	11.3	0.0035	1 vs 2	28	32	8.79	0.6813	NS	K-W	0.231	0.8908	−0.9107	>0.9999	NS
					1 vs 3	28	63	−16.04	0.0952	NS				−3.512	>0.9999	NS
R12B01	K-W	3,78	2.066	0.3559	1 vs 2	32	25	−4.789	0.857	NS	K-W	6.516	0.0385	−8.618	0.3084	NS
					1 vs 3	32	21	−9.04	0.3109	NS				−16.03	0.0235	*
VT057257	K-W	3126	10.88	0.0043	1 vs 2	31	32	−21.74	0.0364	*	K-W	3.119	0.2102	12.92	0.3204	NS
					1 vs 3	31	63	−26.02	0.0023	**				13.53	0.1824	NS
VT038828	K-W	3,62	1.464	0.4808	1 vs 2	26	15	−3.295	>0.9999	NS	K-W	4.462	0.1074	−12.33	0.0701	NS
					1 vs 3	26	21	−6.39	0.4546	NS				−5.159	0.6594	NS
R38G08	K-W	3,83	1.082	0.5821	1 vs 2	26	29	6.631	0.6168	NS	K-W	2.933	0.2307	−6.452	0.6432	NS
					1 vs 3	26	28	2.31	>0.9999	NS				−11.22	0.175	NS
R15B07	K-W	3,85	8.564	0.0138	1 vs 2	29	28	−18.1	0.0113	*	K-W	7.204	0.0273	−9.044	0.3332	NS
					1 vs 3	29	28	−14.3	0.0576	NS				−17.54	0.0146	*
VT042577	K-W	3120	13.2	0.0014	1 vs 2	27	31	−3.256	>0.9999	NS	K-W	7.474	0.0238	21.56	0.0371	*
					1 vs 3	27	62	−24.72	0.0041	**				2.697	>0.9999	NS
R84H09	K-W	3,90	8.832	0.0121	1 vs 2	32	30	−17.27	0.0186	*	K-W	5.947	0.0511	−0.7583	>0.9999	NS
					1 vs 3	32	28	−16.91	0.0248	*				−14.86	0.0559	NS
VT012446	K-W	3120	45.35	<0.0001	1 vs 2	28	29	−7.68	0.8093	NS	K-W	4.805	0.0905	12.13	0.3761	NS
					1 vs 3	28	63	−46.4	<0.0001	****				−4.972	>0.9999	NS
R73A06	K-W	3120	45.9	<0.0001	1 vs 2	25	32	−4.716	>0.9999	NS	K-W	1.651	0.438	11.93	0.3978	NS
					1 vs 3	25	63	−45.6	<0.0001	****				6.513	0.8566	NS
Feb170	K-W	3,80	26.9	<0.0001	1 vs 2	28	24	−29.38	<0.0001	****	K-W	18.19	0.0001	−16.12	0.0253	*
					1 vs 3	28	28	−27.16	<0.0001	****				−26.29	<0.0001	****
R70B05	K-W	3,92	26.38	<0.0001	1 vs 2	28	32	−31.9	<0.0001	****	K-W	12.54	0.0019	−15.88	0.043	*
					1 vs 3	28	32	−30.17	<0.0001	****				−24.21	0.0009	***

△ P(doze)	LP (Fig. 1*E*)										DP (Fig. 1*J*)					
	Nonparametric/parametric test				*Post hoc* comparisons						Nonparametric/parametric test			*Post hoc* comparisons		
Driver	Test	DFn, DFd	*F*	*p*		n1	n2	Mean difference	*p*		Test	*F*	*p*	Mean difference	*p*	
R47F07	K-W	3,95	45.66	<0.0001	1 vs 2	32	31	46.4	<0.0001	****	K-W	23.39	<0.0001	25.36	0.0005	***
					1 vs 3	32	32	16.66	0.0313	*				31.47	<0.0001	****
R28E01	K-W	3,96	15.34	0.0005	1 vs 2	32	32	26.91	0.0002	***	K-W	5.817	0.0545	10.84	0.2389	NS
					1 vs 3	32	32	9.563	0.3394	NS				16.53	0.0352	*
C232	K-W	3,92	12.34	0.0021	1 vs 2	31	31	17.03	0.0241	*	K-W	8.184	0.0167	−2.323	>0.9999	NS
					1 vs 3	31	30	23.11	0.0015	**				15.71	0.0433	*
R70B04	K-W	3,90	21.41	<0.0001	1 vs 2	27	31	30.78	<0.0001	****	K-W	13.43	0.0012	22.41	0.0022	**
					1 vs 3	27	32	9.712	0.3097	NS				21.63	0.0031	**
R53F11	K-W	3,94	62.65	<0.0001	1 vs 2	30	32	54.01	<0.0001	****	K-W	38.71	<0.0001	34.1	<0.0001	****
					1 vs 3	30	32	36.17	<0.0001	****				40.22	<0.0001	****
R56C09	K-W	3,55	7.995	0.0184	1 vs 2	23	11	15.14	0.0199	*	K-W	1.245	0.5367	−4.585	0.87	NS
					1 vs 3	23	21	10.06	0.075	NS				2.06	>0.9999	NS
R54B05	K-W	3,87	16.31	0.0003	1 vs 2	24	31	27.73	0.0001	***	K-W	1.889	0.3889	2.621	>0.9999	NS
					1 vs 3	24	32	15.73	0.0422	*				−5.938	0.768	NS
R38B06	K-W	3,85	20.07	<0.0001	1 vs 2	29	29	26.55	<0.0001	****	K-W	4.584	0.1011	9.897	0.2536	NS
					1 vs 3	29	27	2.854	>0.9999	NS				13.58	0.0793	NS
Aphc507	K-W	3,78	48.18	<0.0001	1 vs 2	28	29	35.39	<0.0001	****	K-W	40.34	<0.0001	31.56	<0.0001	****
					1 vs 3	28	21	39.21	<0.0001	****				36.68	<0.0001	****
R49E12	K-W	3,94	23.26	<0.0001	1 vs 2	31	31	24.03	0.001	**	K-W	0.85	0.6536	−6.226	0.7378	NS
					1 vs 3	31	32	−7.883	0.503	NS				−4.345	>0.9999	NS
R81F01	ANOVA	2,93	12.57	<0.0001	1 vs 2	32	32	0.08838	<0.0001	****	ANOVA	0.488	0.6154	0.03071	0.6904	NS
					1 vs 3	32	32	0.02067	0.5304	NS				0.02308	0.9553	NS
R53G11	ANOVA	2,91	140.8	<0.0001	1 vs 2	31	31	0.4689	<0.0001	****	ANOVA	83.57	<0.0001	0.4356	<0.0001	****
					1 vs 3	31	32	0.4239	<0.0001	****				0.4201	<0.0001	****
VT026841	K-W	3125	35.75	<0.0001	1 vs 2	31	31	13.48	0.2857	NS	K-W	6.354	0.0417	−14.97	0.2077	NS
					1 vs 3	31	63	−30.83	0.0002	***				4.997	>0.9999	NS
VT059775	ANOVA	2110	37.94	<0.0001	1 vs 2	25	25	0.2231	<0.0001	****	ANOVA	7.847	0.0007	−0.00487	>0.9999	NS
					1 vs 3	25	63	0.22	<0.0001	****				0.08162	0.0052	**
R73B05	K-W	3,54	0.229	0.8917	1 vs 2	17	16	−1.706	>0.9999	NS	K-W	4.328	0.1148	−3.813	0.9732	NS
					1 vs 3	17	21	0.7703	>0.9999	NS				6.762	0.3754	NS
R38H02	K-W	3,85	26.52	<0.0001	1 vs 2	27	31	32.8	<0.0001	****	K-W	8.025	0.0181	−15.33	0.0365	*
					1 vs 3	27	27	11.67	0.1649	NS				0.8148	>0.9999	NS
VT040539	K-W	3124	6.565	0.0375	1 vs 2	29	32	14.85	0.214	NS	K-W	4.511	0.1048	12.19	0.3718	NS
					1 vs 3	29	63	−5.067	>0.9999	NS				17.12	0.0675	NS
R64H04	K-W	3,59	4.236	0.1203	1 vs 2	15	23	11.35	0.093	NS	K-W	8.076	0.0176	−15.03	0.0168	*
					1 vs 3	15	21	4.429	0.8913	NS				−4.143	0.9511	NS
R48B10	K-W	3,93	33.55	<0.0001	1 vs 2	30	31	38.6	<0.0001	****	K-W	36.21	<0.0001	35.53	<0.0001	****
					1 vs 3	30	32	28.67	<0.0001	****				36.49	<0.0001	****
R28D01	K-W	3,88	11.65	0.0029	1 vs 2	31	30	10.33	0.2287	NS	K-W	3.733	0.1546	−0.2624	>0.9999	NS
					1 vs 3	31	27	−12.79	0.1143	NS				11.28	0.1871	NS
R41A08	K-W	3,86	15.5	0.0004	1 vs 2	31	28	15.05	0.0415	*	K-W	3.114	0.2108	−4.499	0.9791	NS
					1 vs 3	31	27	−11.35	0.1686	NS				7.286	0.5354	NS
VT042759	K-W	3119	25.96	<0.0001	1 vs 2	26	30	29.2	0.0032	**	K-W	2.782	0.2489	−10.97	0.4702	NS
					1 vs 3	26	63	−9.69	0.4564	NS				−13.3	0.1963	NS
VT045108	K-W	3123	9.84	0.0073	1 vs 2	28	32	16.79	0.1377	NS	K-W	6.609	0.0367	−20.93	0.0466	*
					1 vs 3	28	63	−7.48	0.7112	NS				−3.385	>0.9999	NS
R12B01	K-W	3,77	12.13	0.0023	1 vs 2	31	25	18.97	0.0032	**	K-W	2.735	0.2547	−5	0.8115	NS
					1 vs 3	31	21	16.92	0.0149	*				5.952	0.693	NS
VT057257	K-W	3126	17.41	0.0002	1 vs 2	31	32	35.45	0.0002	***	K-W	25.56	<0.0001	−8.33	0.7308	NS
					1 vs 3	31	63	7.576	0.6886	NS				28.13	0.0009	***
VT038828	K-W	3,62	5.289	0.071	1 vs 2	19	22	12.44	0.0553	NS	K-W	0.959	0.619	5.335	0.6902	NS
					1 vs 3	19	21	9.882	0.1673	NS				4.123	0.9409	NS
R38G08	K-W	3,81	4.512	0.1047	1 vs 2	25	29	3.23	>0.9999	NS	K-W	1.653	0.4377	−7.363	0.503	NS
					1 vs 3	25	27	−9.71	0.2741	NS				−0.7319	>0.9999	NS
R15B07	K-W	3,84	12.6	0.0018	1 vs 2	29	28	−0.351	>0.9999	NS	K-W	1.506	0.4709	−7.621	0.4767	NS
					1 vs 3	29	27	−20.39	0.0035	**				−1.806	>0.9999	NS
VT042577	K-W	3123	25.99	<0.0001	1 vs 2	28	32	21.61	0.0384	*	K-W	4.099	0.1288	−9.924	0.5641	NS
					1 vs 3	28	63	−17.6	0.0595	NS				5.738	0.9571	NS
R84H09	K-W	3,88	39.79	<0.0001	1 vs 2	31	30	38.04	<0.0001	****	K-W	1.079	0.5831	6.716	0.6093	NS
					1 vs 3	31	27	4.256	>0.9999	NS				2.368	>0.9999	NS
VT012446	K-W	3120	21.99	<0.0001	1 vs 2	28	29	43.22	<0.0001	****	K-W	18.98	<0.0001	−17.35	0.1195	NS
					1 vs 3	28	63	21.6	0.0125	*				16.15	0.0819	NS
R73A06	K-W	3120	1.409	0.4943	1 vs 2	25	32	−6.146	>0.9999	NS	K-W	1.733	0.4204	−10.97	0.4748	NS
					1 vs 3	25	63	−9.716	0.4747	NS				−2.466	>0.9999	NS
Feb170	K-W	3,81	21.58	<0.0001	1 vs 2	29	25	28.27	<0.0001	****	K-W	11.39	0.0034	−10.35	0.2139	NS
					1 vs 3	29	27	21.31	0.0014	**				11.65	0.128	NS
R70B05	ANOVA	2,86	23.94	<0.0001	1 vs 2	27	30	0.2709	<0.0001	****	ANOVA	69.04	<0.0001	0.4665	<0.0001	****
					1 vs 3	27	32	0.1498	0.0004	***				0.4365	<0.0001	****

△ P(wake)	LP (Fig. 1*F*)										DP (Fig. 1*K*)					
	Nonparametric/parametric test				*Post hoc* comparisons						Nonparametric/parametric test			*Post hoc* comparisons		
Driver	Test	DFn, DFd	*F*	*p*		n1	n2	Mean difference	*p*		Test	*F*	*p*	Mean difference	*p*	
R47F07	K-W	3,95	20.67	<0.0001	1 vs 2	32	31	−22.98	0.0019	**	K-W	33.22	<0.0001	21.38	0.0042	**
					1 vs 3	32	32	−29.97	<0.0001	****				39.69	<0.0001	****
R28E01	K-W	3,96	21.46	<0.0001	1 vs 2	32	32	−31.44	<0.0001	****	K-W	9.154	0.0103	20.5	0.0065	**
					1 vs 3	32	32	−9.438	0.3507	NS				6.031	0.7729	NS
C232	K-W	3,92	5.439	0.0659	1 vs 2	31	31	−10.77	0.2243	NS	K-W	0.371	0.8309	−0.1935	>0.9999	NS
					1 vs 3	31	30	−15.53	0.0464	*				−3.708	>0.9999	NS
R70B04	K-W	3,90	24.58	<0.0001	1 vs 2	27	31	−32.25	<0.0001	****	K-W	1.492	0.4741	7.388	0.5653	NS
					1 vs 3	27	32	−7.976	0.4854	NS				0.603	>0.9999	NS
R53F11	K-W	3,94	31.47	<0.0001	1 vs 2	30	32	−18.44	0.0156	*	K-W	5.14	0.0765	15.13	0.0582	NS
					1 vs 3	30	32	19.81	0.0085	**				4.16	>0.9999	NS
R56C09	K-W	3,55	0.134	0.9352	1 vs 2	23	11	1.277	>0.9999	NS	K-W	1.029	0.5978	5.387	0.718	NS
					1 vs 3	23	21	−0.8965	>0.9999	NS				3.669	0.896	NS
R54B05	K-W	3,87	3.497	0.174	1 vs 2	24	31	−11.23	0.2039	NS	K-W	16.74	0.0002	27.92	<0.0001	****
					1 vs 3	24	32	−1.24	>0.9999	NS				13.17	0.1071	NS
R38B06	K-W	3,85	22.1	<0.0001	1 vs 2	29	29	−29.45	<0.0001	****	K-W	3.352	0.1871	5.931	0.7203	NS
					1 vs 3	29	27	−7.777	0.4775	NS				12.08	0.1343	NS
Aphc507	K-W	3,78	7.019	0.0299	1 vs 2	28	29	−7.612	0.4097	NS	K-W	35.78	<0.0001	32.48	<0.0001	****
					1 vs 3	28	21	9.583	0.2858	NS				31.29	<0.0001	****
R49E12	K-W	3,94	13.47	0.0012	1 vs 2	31	31	−12.16	0.1585	NS	K-W	10.84	0.0044	18.94	0.0126	*
					1 vs 3	31	32	13.06	0.1149	NS				20.36	0.0061	**
R81F01	K-W	3,96	11.28	0.0036	1 vs 2	32	32	0.5	>0.9999	NS	K-W	13.12	0.0014	22.13	0.003	**
					1 vs 3	32	32	20.5	0.0065	**				0.5625	>0.9999	NS
R53G11	K-W	3,94	10.85	0.0044	1 vs 2	31	31	4.968	0.9468	NS	K-W	25.01	<0.0001	34.29	<0.0001	****
					1 vs 3	31	32	21.58	0.0034	**				12.88	0.1219	NS
VT026841	K-W	3125	12.64	0.0018	1 vs 2	31	31	−27.65	0.0053	**	K-W	1.709	0.4254	−10.32	0.5239	NS
					1 vs 3	31	63	−1.502	>0.9999	NS				−0.809	>0.9999	NS
VT059775	K-W	3113	29.41	<0.0001	1 vs 2	25	25	7	0.9001	NS	K-W	2.853	0.2402	−9.72	0.5885	NS
					1 vs 3	25	63	36.83	<0.0001	****				−13.07	0.1827	NS
R73B05	K-W	3,54	4.271	0.1182	1 vs 2	17	16	−5.555	0.6214	NS	K-W	5.594	0.061	12.86	0.0379	*
					1 vs 3	17	21	5.216	0.6191	NS				7.521	0.2857	NS
R38H02	K-W	3,85	2.746	0.2533	1 vs 2	27	31	9.661	0.274	NS	K-W	2.862	0.2391	0.5317	>0.9999	NS
					1 vs 3	27	27	9.37	0.3261	NS				10	0.2731	NS
VT040539	K-W	3124	33.57	<0.0001	1 vs 2	29	32	−13.52	0.2846	NS	K-W	4.112	0.128	18.66	0.0858	NS
					1 vs 3	29	63	29.09	0.0006	***				10.51	0.3848	NS
R64H04	K-W	3,59	5.863	0.0533	1 vs 2	15	23	−4.896	0.7808	NS	K-W	4.294	0.1169	10.76	0.1182	NS
					1 vs 3	15	21	7.61	0.38	NS				10.5	0.1408	NS
R48B10	K-W	3,93	19.43	19.43	1 vs 2	30	31	16.61	0.0325	*	K-W	1.99	0.3698	2.994	>0.9999	NS
					1 vs 3	30	32	30.21	<0.0001	****				−6.388	0.7035	NS
R28D01	K-W	3,88	16.8	0.0002	1 vs 2	31	30	−20.47	0.0035	**	K-W	5.059	0.0797	5.797	0.7513	NS
					1 vs 3	31	27	5.559	0.8169	NS				15.06	0.0503	NS
R41A08	K-W	3,86	3.179	0.204	1 vs 2	31	28	6.783	0.5948	NS	K-W	5.348	0.069	14.63	0.0492	*
					1 vs 3	31	27	11.61	0.1545	NS				10.1	0.2486	NS
VT042759	K-W	3119	8.507	0.0142	1 vs 2	26	30	−1.505	>0.9999	NS	K-W	0.097	0.9526	−1.413	>0.9999	NS
					1 vs 3	26	63	17.64	0.0564	NS				0.9634	>0.9999	NS
VT045108	K-W	3123	20.09	<0.0001	1 vs 2	28	32	−6.754	0.9282	NS	K-W	8.545	0.014	11.15	0.4535	NS
					1 vs 3	28	63	24.84	0.0043	**				23.06	0.0088	**
R12B01	K-W	3,77	12.28	0.0022	1 vs 2	31	25	−3.947	>0.9999	NS	K-W	2.253	0.3241	7.408	0.436	NS
					1 vs 3	31	21	17.95	0.0091	**				−1.604	>0.9999	NS
VT057257	K-W	3126	15.43	0.0004	1 vs 2	31	32	−2.342	>0.9999	NS	K-W	12.62	0.0018	−27.62	0.0054	**
					1 vs 3	31	63	24.32	0.0048	**				−26.39	0.002	**
VT038828	K-W	3,62	3.449	0.1783	1 vs 2	19	22	−1.543	>0.9999	NS	K-W	3.602	0.1651	9.1	0.2145	NS
					1 vs 3	19	21	8.065	0.316	NS				0.02256	>0.9999	NS
R38G08	K-W	3,81	5.615	0.0604	1 vs 2	25	29	−7.476	0.4886	NS	K-W	5.318	0.07	10.32	0.2157	NS
					1 vs 3	25	27	7.43	0.5104	NS				14.71	0.0485	*
R15B07	K-W	3,84	5.756	0.0562	1 vs 2	29	28	−5.112	0.8579	NS	K-W	9.158	0.0103	1.052	>0.9999	NS
					1 vs 3	29	27	10.4	0.2219	NS				17.74	0.0131	*
VT042577	K-W	3123	23.35	<0.0001	1 vs 2	28	32	−7.221	0.8676	NS	K-W	31.86	<0.0001	28.4	0.0042	**
					1 vs 3	28	63	26.82	0.0019	**				45.57	<0.0001	****
R84H09	K-W	3,88	19.71	<0.0001	1 vs 2	31	30	21.25	0.0023	**	K-W	27.23	<0.0001	28.34	<0.0001	****
					1 vs 3	31	27	28.32	<0.0001	****				31.12	<0.0001	****
VT012446	K-W	3120	47.36	<0.0001	1 vs 2	28	29	2.543	>0.9999	NS	K-W	39.32	<0.0001	27.13	0.0065	**
					1 vs 3	28	63	45.02	<0.0001	****				49.01	<0.0001	****
R73A06	K-W	3120	77.72	<0.0001	1 vs 2	25	32	−4.016	>0.9999	NS	K-W	15.11	0.0005	10.21	0.5429	NS
					1 vs 3	25	63	53.74	<0.0001	****				29.44	0.0007	***
Feb170	K-W	3,81	21.73	<0.0001	1 vs 2	29	25	26.48	<0.0001	****	K-W	52.79	<0.0001	33.97	<0.0001	****
					1 vs 3	29	27	24.31	0.0002	***				43.34	<0.0001	****
R70B05	K-W	3,89	29.88	<0.0001	1 vs 2	27	30	30.76	<0.0001	****	K-W	53.64	<0.0001	45.1	<0.0001	****
					1 vs 3	27	32	34	<0.0001	****				42.08	<0.0001	****

*^a^*Change in sleep parameters for total sleep, number of episodes, maximum episode length, P(doze), and P(wake) on the activation day (30°C) were analyzed for day (LP) and night (DP) separately. Datasets that had a normal distribution, one-way ANOVA followed by Bonferroni test was applied. For datasets that did not pass the normality test, Kruskal–Wallis (K-W) followed by Dunn's test was applied. *Post hoc* tests were applied between the experimental group (F1 generation of the cross of GAL4 lines to UAS-dTrpA1)(1) and the genetic control groups (F1 generation of the crosses of either GAL4 lines to w^CS^ or UAS-dTrpA1 to w^CS^)(2 or 3).

*p < 0.05.

**p < 0.01.

***p < 0.001.

****p < 0.0001.

We also found cell groups which, when activated, induced a significant reduction in total sleep: Feb170-GAL4^+^ and R70B05-GAL4^+^ neurons (Fig. 1*B*; Table 1). Sleep reduction was associated with significant decreases in maximum episode length with no change in the number of episodes compared with their genetic controls (Fig. 1*C*,*D*; Table 1). The reduced sleep amount and episode length were possibly because of the increased P(doze) and P(wake) (Fig. 1*E*,*F*; Table 1), suggesting neurons labeled by these two drivers are involved in upregulation of sleep pressure and downregulation of sleep depth during the daytime.

Nighttime effects of thermogenetic neuron activation are more complex to interpret. Data have to be viewed in the context of the sleep-suppressing effects of elevated temperature on normal WT animal sleep (Parisky et al., 2016; Jin et al., 2021). This temperature effect can be visualized in the continuous sleep plots for most of the GAL4 and UAS control lines in Figures 2, 3, and 6. VT059775-GAL4^+^ and VT057257-GAL4^+^ neuron activation led to almost no change of total sleep compared with their own baseline, but this reflects a significant difference from genetic controls, which respond to heat with at large reduction in sleep. These lines also had only small reductions in P(wake) compared with controls, implying that these neurons may be involved in sleep promotion by changing sleep depth (Fig. 1*G*,*K*; Table 1).

We also found a number of GAL4 drivers, including R47F07, Aphc507, R64H04, R84H09, Feb170, and R70B05 which significantly reduced nighttime sleep amount compared with their controls, suggesting they contribute to promoting wakefulness (Fig. 1*G*; Table 1). These reductions in total sleep were accompanied by changes in sleep structure, featured as fragmentation where the number of episodes significantly increased and/or episode length reduced (Fig. 1*H*,*I*; Table 1). Many drivers exhibited increased P(doze) and P(wake) (Fig. 1*J*,*K*; Table 1), suggesting sleep pressure and sleep depth play important roles in nighttime sleep.

### Thermoactivation of ring neurons can change sleep structure independent of sleep amount

We also found cases where sleep structure was changed without alterations in total sleep, supporting the idea that structure can be regulated independently (C. Liu et al., 2019). Activation of neurons from several GAL4 drivers, including R70B04, R53F11, R54B05, R53G11, R48B10, and VT038828, resulted in significant change only in sleep structure. Except for R70B04, which induced consolidated daytime sleep with a decrease in the number of episodes and an increase in the episode length, all drivers mentioned above exhibited fragmented sleep either during the day or at night (Fig. 1*C*,*D*,*H*,*I*; Table 1). Fragmentation was accompanied by a robust increase in P(doze) for the majority drivers (Fig. 1*E*,*J*; Table 1). P(doze) is believed to correlate with sleep pressure (Wiggin et al., 2020), suggesting the fragmentation reflects an increase in the probability of switching from wake to sleep (i.e., high sleep drive rather than from an inability to maintain the sleep state).

The circadian period during which fragmentation occurred varied with GAL4 line. Daytime fragmentation was observed when R54B05-GAL4^+^ and R48B10-GAL4^+^ neurons were activated (Fig. 1*C*; Table 1), and nighttime fragmentation was seen when VT038828-GAL4^+^ neurons were activated (Fig. 1*H*; Table 1). Fragmentation of both day and night was found when R53G11-GAL4^+^ and R53F11-GAL4^+^ neurons were activated (Fig. 1*C*,*D*,*H*,*I*; Table 1).

The structural parameters that were altered were also variable. Three GAL4 drivers, R53F11, R54B05, and VT038828, only exhibited a significant increase in the number of episodes. R53G11 and R48B10 only showed reduced episode length. All of these changes contributed to increases in P(doze) with little or weak P(wake) effects, especially during the day (Fig. 1*E*,*F*,*J*,*K*; Table 1). Interestingly, R12B01-GAL4 did not exhibit detectable changes in the number of episodes or episode length, but had a significant increase in P(doze) compared with both controls (Fig. 1*E*; Table 1), suggesting a potential specific contribution of R28E01-GAL4^+^ neurons to control of sleep pressure. Together, changes in sleep structure are highly associated with P(doze), but when sleep structure changes are accompanied by changes in total sleep amount, P(wake) becomes an important component of the regulation.

### Thermoactivation of ring neurons has complex effects on sleep homeostasis

We summarized drivers with significant changes of total amount of sleep or sleep structure during the day, at night, or both (Fig. 2*A*). We plotted sleep and changes in parameters over 3 d to provide a more nuanced picture of the lasting effects of activation of these neurons and present the lines ordered from largest to smallest rebound sleep on the recovery day (Figs. 2*B–Q*, 3; Tables 2–4). For some of the lines, the changes in total sleep appeared to activate homeostatic changes that were evident during the recovery day. Activation of C232-GAL4^+^ neurons, which increases sleep on the activation day, leads to a negative rebound (decrease in sleep) on cessation of activation (Fig. 2*C*; Tables 2 and 3). Activation of Feb170-GAL4^+^ neurons decreased sleep both in the day and night, and this was followed by a homeostatic rebound increase in sleep (Fig. 2*G*; Tables 2 and 3). Activation of R48B10-GAL4^+^ or R53F11-GAL4^+^ neurons led to fragmentation during either the day or both in the day and night, and a robust homeostatic rebound increase occurred (Figs. 2*J–Q*; Tables 2 and 3). Interestingly, some drivers exhibited decreased sleep without a rebound change in sleep afterward (e.g., R64H04, R47F07, and R84H09; Fig. 3; Table 4), suggesting that, for these lines, sleep loss was either not able to be compensated for or was not “counted” by the homeostat. These may represent cell types that are not integrated into the homeostat (Seidner et al., 2015).

**Table 2.** Power analysis for the sample size of 4 drivers used in Figure 2*^a^*

Total sleep	Experiment vs GAL4 Control				Experiment vs UAS Control			
	30°C		21°C		30°C		21°C	
Drivers	LP	DP	LP	DP	LP	DP	LP	DP
c232	1	0.167	0.377	0.639	1	0.563	0.533	0.907
Feb170	1	1	0.946	0.209	1	1	0.996	0.19
R48B10	0.151	0.53	0.934	0.077	1	0.998	0.975	0.999
R53F11	1	0.052	0.999	0.858	0.108	0.996	0.85	0.928

No. of episodes	Experiment vs GAL4 Control				Experiment vs UAS Control			
	30°C		21°C		30°C		21°C	
Drivers	LP	DP	LP	DP	LP	DP	LP	DP
c232	0.057	0.09	0.497	0.268	0.102	0.347	0.561	0.186
Feb170	0.929	0.99	0.234	0.083	0.819	0.06	0.989	0.86
R48B10	1	0.977	0.985	0.286	1	0.948	0.521	0.287
R53F11	0.816	1	0.948	0.149	0.705	0.992	0.323	0.078

Maximum episode length	Experiment vs GAL4 Control				Experiment vs UAS Control			
	30°C		21°C		30°C		21°C	
Drivers	LP	DP	LP	DP	LP	DP	LP	DP
c232	0.999	0.182	0.075	0.05	0.975	0.46	0.159	0.057
Feb170	1	0.795	0.053	0.05	0.993	0.859	0.673	0.121
R48B10	0.814	0.051	0.244	0.061	1	0.21	0.502	0.086
R53F11	0.815	0.188	0.275	0.219	0.413	0.091	0.19	0.054

*^a^*Four GAL4 drivers were included in the analysis: c232, Feb170, R48B10, and R53F11. The experimental group was compared with either GAL4 control group or UAS-dTrpA1 control group for both activation day and recovery day. Total sleep, number of episodes, and maximum episode length for LP and DP were analyzed separately. Power analysis was conducted using the script of sampsizepwr in MATLAB.

**Table 3.** Statistical analysis of the recovery day for 4 drivers used in Figure 2*^a^*

	LP										DP					
Driver	Nonparametric/parametric test				*Post hoc* comparisons						Nonparametric/parametric test			*Post hoc* comparisons		
D3-D1 21°C	Test	DFn, DFd	*F*	*p*		n1	n2	Mean difference	*p*		Test	*F*	*p*	Mean difference	*p*	
C232																
△ Total sleep	K-W	3,91	4.103	0.1285	1 vs 2	30	31	8.725	0.3943	NS	K-W	16.77	0.0002	−21.16	0.0035	**
					1 vs 3	30	30	13.63	0.0912	NS				−26.32	0.0002	***
△ No. of episodes	K-W	3,91	4.451	0.108	1 vs 2	30	31	8.845	0.3803	NS	K-W	2.186	0.3352	9.984	0.2786	NS
					1 vs 3	30	30	14.22	0.0735	NS				4.9	0.9435	NS
△ Maximum episode length	K-W	3,91	1.39	0.4991	1 vs 2	30	31	−2.339	>0.9999	NS	K-W	0.326	0.8494	−1.673	>0.9999	NS
					1 vs 3	30	30	5.45	0.8483	NS				2.183	>0.9999	NS
△ P(doze)	K-W	3,92	28.85	<0.0001	1 vs 2	31	31	−30.23	<0.0001	****	K-W	4.809	0.0903	−13.87	0.0817	NS
					1 vs 3	31	30	2.694	>0.9999	NS				−2.237	>0.9999	NS
△ P(wake)	K-W	3,92	5.069	0.0793	1 vs 2	31	31	−13.42	0.0957	NS	K-W	10.1	0.0064	17.19	0.0225	*
					1 vs 3	31	30	−13.09	0.1112	NS				19.97	0.007	**
Feb170																
△ Total sleep	K-W	3,80	27.64	<0.0001	1 vs 2	28	24	15.15	0.0382	*	K-W	3.219	0.2	−10.73	0.1939	NS
					1 vs 3	28	28	32.63	<0.0001	****				−8.661	0.3263	NS
△ No. of episodes	ANOVA	2,77	9.999	0.0001	1 vs 2	28	24	2.488	0.1756	NS	ANOVA	4.401	0.0155	1.405	0.7464	NS
					1 vs 3	28	28	7.679	<0.0001	****				5.964	0.0108	*
△ Maximum episode length	K-W	3,80	8.277	0.0159	1 vs 2	28	24	0.6994	>0.9999	NS	K-W	0.452	0.7979	−0.7917	>0.9999	NS
					1 vs 3	28	28	15.98	0.0201	*				−3.964	>0.9999	NS
△ P(doze)	K-W	3,81	44.43	<0.0001	1 vs 2	29	25	18.76	0.007	**	K-W	18.67	<0.0001	11.07	0.1695	NS
					1 vs 3	29	27	41.91	<0.0001	****				27.1	<0.0001	****
△ P(wake)	K-W	3,81	11.13	0.0038	1 vs 2	29	25	0.06207	>0.9999	NS	K-W	10.96	0.0042	19.4	0.005	**
					1 vs 3	29	27	−18.47	0.0067	**				16.48	0.0176	*
R48B10																
△ Total sleep	K-W	3,95	17.59	0.0002	1 vs 2	31	32	23.65	0.0013	**	K-W	0.76	0.684	−5.828	0.803	NS
					1 vs 3	31	32	26.67	0.0002	***				−1.546	>0.9999	NS
△ No. of episodes	K-W	3,95	11.89	0.0026	1 vs 2	31	32	23.91	0.0011	**	K-W	4.2	0.1224	11.79	0.1781	NS
					1 vs 3	31	32	12	0.167	NS				12.82	0.1289	NS
△ Maximum episode length	K-W	3,95	2.567	0.2771	1 vs 2	31	32	8.717	0.4192	NS	K-W	1.002	0.606	−3.074	>0.9999	NS
					1 vs 3	31	32	10.39	0.2696	NS				−6.933	0.6365	NS
△ P(doze)	ANOVA	2,90	3.153	0.0475	1 vs 2	30	31	0.02628	0.4415	NS	ANOVA	13.58	<0.0001	0.1613	0.0003	***
					1 vs 3	30	32	0.05309	0.0277	*				0.2017	<0.0001	****
△ P(wake)	K-W	3,93	8.553	0.0139	1 vs 2	30	31	−16.77	0.0305	*	K-W	2.183	0.3357	10.15	0.2844	NS
					1 vs 3	30	32	−18.15	0.0163	*				6.156	0.7389	NS
R53F11																
△ Total sleep	K-W	3,95	15.56	0.0004	1 vs 2	31	32	23.22	0.0017	**	K-W	9.302	0.0096	20.46	0.0064	**
					1 vs 3	31	32	24.33	0.0009	***				5.681	0.8266	NS
△ No. of episodes	K-W	3,95	7.201	0.0273	1 vs 2	31	32	18.6	0.0147	*	K-W	1.607	0.4479	−7.84	0.5069	NS
					1 vs 3	31	32	10.08	0.2921	NS				−0.7308	>0.9999	NS
△ Maximum episode length	K-W	3,95	1.743	0.4183	1 vs 2	31	32	−8.788	0.4117	NS	K-W	2.279	0.3201	8.549	0.4369	NS
					1 vs 3	31	32	−6.726	0.6659	NS				−0.8881	>0.9999	NS
△ P(doze)	ANOVA	2,95	1.461	0.2371	1 vs 2	30	34	0.01617	0.8543	NS	K-W	9.719	0.0078	9.725	0.3443	NS
					1 vs 3	30	34	0.03458	0.183	NS				22.08	0.0039	**
△ P(wake)	K-W	3,98	9.365	0.0093	1 vs 2	30	34	−19.12	0.0146	*	K-W	5.342	0.0692	−15.8	0.0531	NS
					1 vs 3	30	34	−19.03	0.0151	*				−4.475	>0.9999	NS

*^a^*Change in sleep parameters, total sleep, number of episodes, maximum episode length, P(doze), and P(wake) on the recovery day (21°C) were analyzed for day (LP) and night (DP) separately. One-way ANOVA followed by Bonferroni test or Kruskal–Wallis (K-W) followed by Dunn's test was applied based on distribution of the datasets.

*p < 0.05.

**p < 0.01.

***p < 0.001.

****p < 0.0001.

**Table 4.** Statistical analysis of the recovery day for 3 drivers used in Figure 3*^a^*

	LP										DP					
Driver	Nonparametric/parametric test				*Post hoc* comparisons						Nonparametric/parametric test			*Post hoc* comparisons		
D3-D1 21°C	Test	DFn, DFd	*F*	*p*		n1	n2	Mean difference	*p*		Test	*F*	*p*	Mean difference	*p*	
R64H04																
△ Total sleep	ANOVA	2,55	5.451	0.0069	1 vs 2	15	22	−121.1	0.0035	**	ANOVA	2.027	0.1415	−29.82	0.6373	NS
					1 vs 3	15	21	−85.58	0.0464	*				−75.3	0.0959	NS
△ No. of episodes	ANOVA	2,55	0.0772	0.9258	1 vs 2	15	22	−0.5091	0.9676	NS	ANOVA	0.098	0.9072	−1.006	0.9253	NS
					1 vs 3	15	21	−0.981	0.8884	NS				−1.4	0.8643	NS
△ Maximum episode length	K-W	3,58	8.367	0.0152	1 vs 2	15	22	−16.35	0.0077	**	K-W	2.913	0.2331	−9.277	0.2017	NS
					1 vs 3	15	21	−9.386	0.2003	NS				−7.681	0.3569	NS
△ P(doze)	ANOVA	2,56	3.591	0.0341	1 vs 2	15	23	−0.02696	0.7393	NS	K-W	1.256	0.2927	−0.07932	0.2415	NS
					1 vs 3	15	21	0.04529	0.2829	NS				−0.05507	0.5749	NS
△ P(wake)	ANOVA	2,56	5.536	0.0064	1 vs 2	15	23	0.1066	0.016	*	K-W	3.68	0.1588	0.5768	>0.9999	NS
					1 vs 3	15	21	0.124	0.0054	**				9.295	0.2188	NS
R47F07																
△ Total sleep	ANOVA	2,92	7.799	0.0007	1 vs 2	24	39	−107.8	0.001	**	ANOVA	12.99	<0.0001	−54.83	0.0075	**
					1 vs 3	24	32	−25.5	0.6125	NS				30.61	0.1952	NS
△ No. of episodes	K-W	3,95	9.574	0.0083	1 vs 2	24	39	12.56	0.1576	NS	K-W	2.283	0.3194	−10.53	0.2796	NS
					1 vs 3	24	32	−7.448	0.633	NS				−4.609	>0.9999	NS
△ Maximum episode length	K-W	3,95	13.04	0.0015	1 vs 2	24	39	−20.25	0.0093	**	K-W	0.042	0.9793	−0.00641	>0.9999	NS
					1 vs 3	24	32	0.8698	>0.9999	NS				−1.229	>0.9999	NS
△ P(doze)	K-W	3,95	3.265	0.1954	1 vs 2	32	31	12.13	0.1615	NS	K-W	5.942	0.0513	−5.134	0.9198	NS
					1 vs 3	32	32	8.75	0.4085	NS				11.38	0.1977	NS
△ P(wake)	ANOVA	2,92	2.319	0.1041	1 vs 2	32	31	0.07036	0.1217	NS	K-W	9.721	0.0077	6.953	0.6339	NS
					1 vs 3	32	32	−0.00178	0.9983	NS				−14.25	0.0774	NS
R84H09																
△ Total sleep	K-W	3,90	0.3417	0.8429	1 vs 2	32	30	−2.221	>0.9999	NS	K-W	6.194	0.0452	10.71	0.2137	NS
					1 vs 3	32	28	1.777	>0.9999	NS				−6.096	0.7344	NS
△ No. of episodes	K-W	3,90	0.934	0.6269	1 vs 2	32	30	−0.725	>0.9999	NS	K-W	1.779	0.4108	1.976	>0.9999	NS
					1 vs 3	32	28	−6.054	0.7396	NS				8.681	0.3972	NS
△ Maximum episode length	K-W	3,90	1.147	0.5634	1 vs 2	32	30	−2.349	>0.9999	NS	K-W	1.838	0.399	1.258	>0.9999	NS
					1 vs 3	32	28	4.877	0.9412	NS				−7.375	0.5506	NS
△ P(doze)	K-W	3,88	8.562	0.0138	1 vs 2	31	30	12.25	0.1223	NS	K-W	2.214	0.3305	−1.055	>0.9999	NS
					1 vs 3	31	27	−7.252	0.5617	NS				8.217	0.4435	NS
△ P(wake)	K-W	3,88	0.9652	0.6172	1 vs 2	31	30	6.324	0.6676	NS	K-W	3.736	0.1545	−9.147	0.3242	NS
					1 vs 3	31	27	2.068	>0.9999	NS				3.382	>0.9999	NS

*^a^*Change in sleep parameters, total sleep, number of episodes, maximum episode length, P(doze), and P(wake) on the recovery day (21°C) were analyzed for day (LP) and night (DP) separately. One-way ANOVA followed by Bonferroni test or Kruskal–Wallis (K-W) followed by Dunn's test was applied based on distribution of the datasets.

*p < 0.05.

**p < 0.01.

### Association of changes in arousal and sleep drive with GAL4^+^ groups of ring neurons

**Figure 4.**
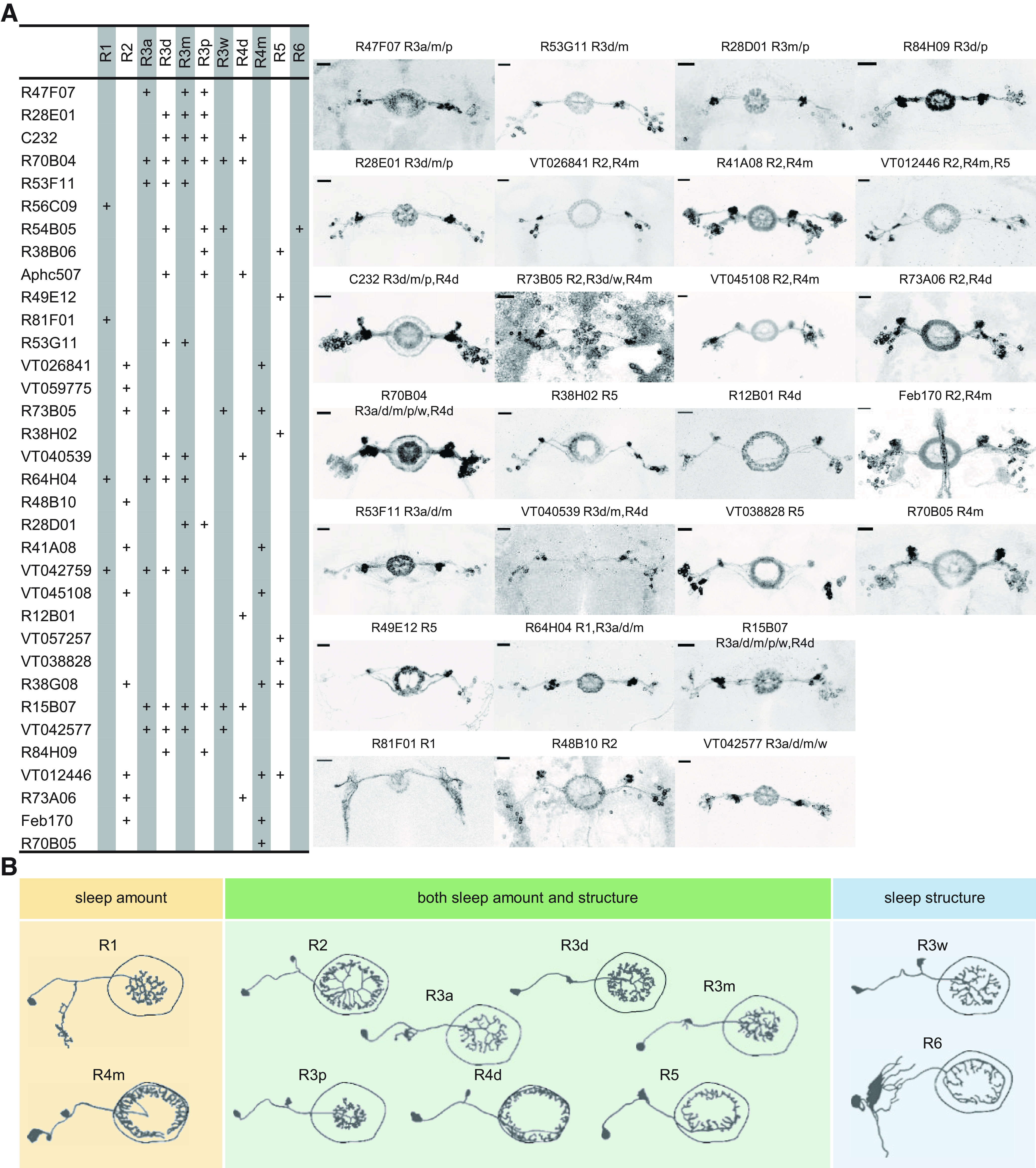
Expression patterns of EB drivers in the screen. ***A***, Distinct subtypes of ring neurons were labeled by the 34 drivers. Expression patterns of all the publicly available drivers (26) are shown. ***B***, Single subtype of ring neurons that are involved in the regulation of sleep amount (yellow), structure (blue), and both amount and structure (green). “+” in columns indicates where the driver has expression. Scale bar: 20 μm.

The majority of the GAL4 lines we screened contained more than one subtype of ring neuron (Fig. 4*A*), and exhibited expression outside the EB in other areas of the central brain (Table 5). To examine the linkage between ring neuron types and distinct aspects of sleep amount and/or sleep structure, we first separated drivers into two groups (Fig. 2*A*): (1) those that exhibited changes in sleep amount and (2) those that exhibited no change in sleep amount but had changes in sleep structure. Based on the time of day when the phenotype was observed (day only, night only, or both day and night), we classed those drivers into three clusters. For lines that changed total sleep, we noted their effects in Figure 2*A* as increasing or decreasing. The second type of information we layered into the analysis was the identification of the subtypes of ring neurons in each line according to anatomic features and recent nomenclature (Omoto et al., 2018; Hulse et al., 2021) (Fig. 4*A*). Based on this primary classification, many subtypes of ring neurons, including R1, R2, R4m, R4d, R5, and many R3 subtypes (R3a, R3m, R3d, and R3p), may participate in the regulation of sleep amount (Fig. 4*B*). Because of the multiplicity of ring neurons in these EB drivers, it was hard to *a priori* link a single subtype of EB neuron with a specific function in the regulation of sleep amount/structure. Thus, we used statistical models to try to identify links between ring subtypes and phenotypes.

**Table 5.** Expression patterns of 34 drivers outside the EB in the central brain*^a^*

	Sleep Amount	Sleep Structure	AL	AMMC	AOTU	ATL	AVLP	CL	FB	GA	GNG	ICL	LH	LO	LOP	MB	ME	NO	OL	PI	PRW	SAD	SCL	SEZ	SIP	SLP	SMP	WED	Ventrolateral protocerebrum	Adult pheromone projection PPN1 neuron	Large field neuron	Source
R47F07	√	√									+			+	+		+				+											VF
R28E01	√																															FB
C232	√																														+	FB
R70B04		√																														FB
R53F11		√																														FB
R56C09	√																													+		FB
R54B05		√	+						+		+		+	+							+							+				VF
R38B06									+	+		+																				FB
Aphc507	√	√																		+											+	FB
R49E12																																FB
R81F01																																VF
R53G11		√	+		+		+		+		+		+	+		+	+				+	+			+	+	+					VF
VT026841			+					+	+							+		+						+	+		+		+			FB
VT059775	√	√	+			+		+	+				+			+	+	+						+	+	+	+		+			FB
R73B05							+				+																					VF
R38H02			+													+																FB
VT040539			+					+	+							+		+							+		+					FB
R64H04	√																															FB
R48B10		√	+		+																+											FB
R28D01																																FB
R41A08					+																											FB
VT042759								+	+							+		+							+		+		+			FB
VT045108			+					+	+							+		+							+		+		+			FB
R12B01																																FB
VT057257	√		+	+	+			+	+							+									+	+	+		+			FB
VT038828		√						+	+							+									+		+		+			FB
R38G08																																FB
R15B07																																FB
VT042577								+	+							+		+						+	+		+		+			FB
R84H09	√										+											+						+				VF
VT012446					+	+		+	+							+		+					+		+	+	+		+			FB
R73A06																																FB
Feb170	√								+										+													FB
R70B05	√				+		+				+			+			+				+	+										VF

*^a^*Drivers are listed in the first column. √ (in the second or third column) indicates whether they had a phenotype for sleep amount and/or sleep structure. + (in subsequent columns) indicates where the driver has expression. The regions of the central brain were in abbreviation based on description of FlyBase and Virtual FlyBrain in alphabet order. AL, Antennal lobe; AMMC, antennal mechanosensory and motor center; AOTU, anterior optic tubercle; ATL, antler; AVLP, anterior ventrolateral protocerebrum; CL, clamp; GA, gall; GNG, gnathal ganglion; ICL, inferior clamp; LH, lateral horn; LO, lobula; LOP, lobula plate; ME, medulla; NO, nodulus; OL, optic lobe; PI, pars intercerebralis; PRW, prow; SAD, saddle; SCL, superior clamp; SEZ, subesophageal zone; SIP, superior intermediate protocerebrum; SLP, superior lateral protocerebrum; SMP, superior medial protocerebrum; WED, wedge. Source of images for expression analysis is listed in the last column. VF, virtual fly brain; FB, FlyBase. Non-EB expression was not predictive of either total sleep or sleep structure phenotypes.

**Figure 5.**
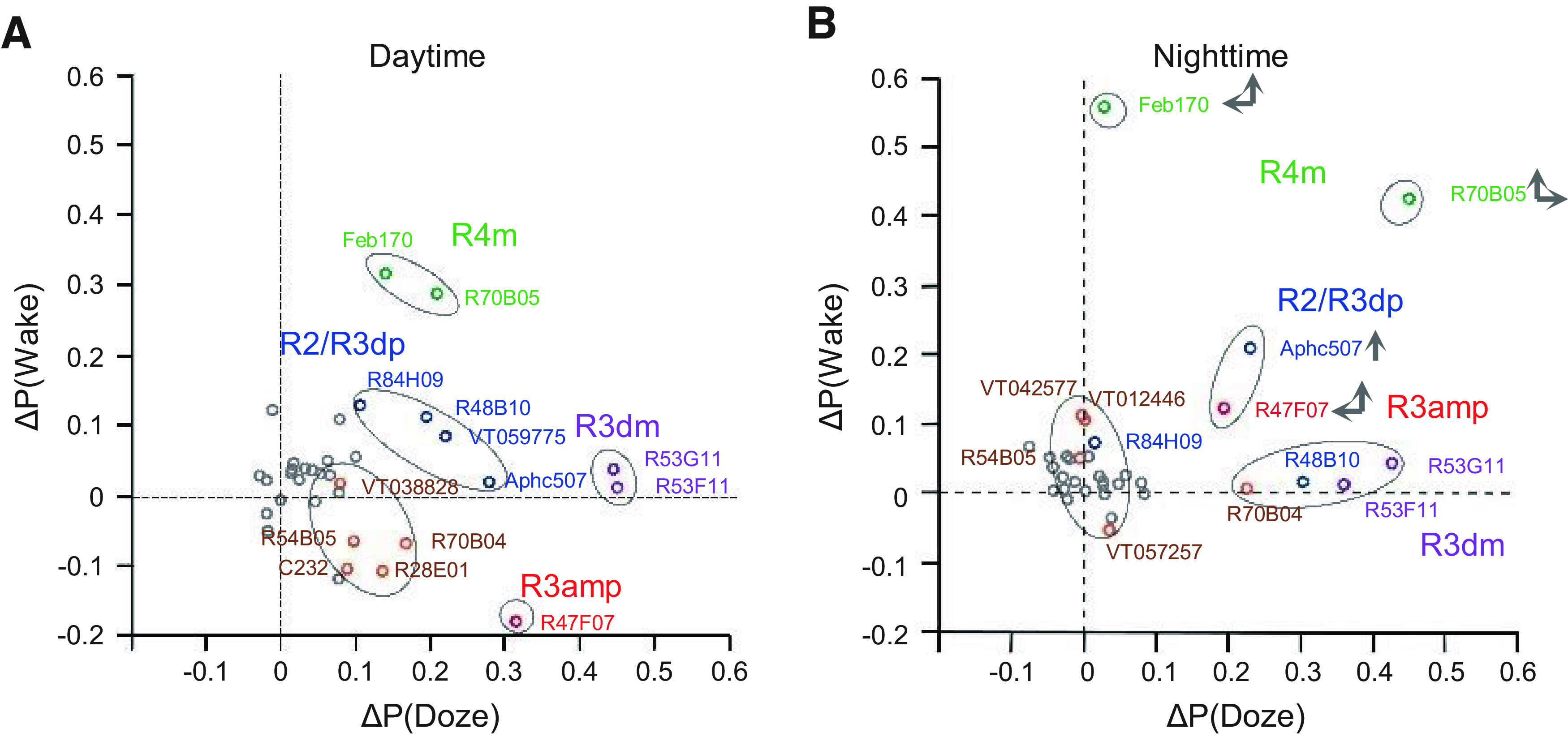
Association of changes in arousal and sleep drive with GAL4^+^ groups of ring neurons. Mixed Gaussian model cluster analysis for drivers have similar patterns during the daytime (***A***) and at night (***B***). Gray dots represent activation did not show significance in P(wake)/P(doze) analysis. Green dots represent increase in both P(wake) and P(doze). Blue dots represent mild increase in both P(wake) and P(doze). Brown dots represent weak increase in both P(wake) and P(doze). Purple dots represent increase only in P(doze). Red dots represent increase in P(doze) and decrease in P(wake). Vertical and horizontal arrows in nighttime panel represent shifts in location of P(doze) and P(wake) compared with the daytime.

**Figure 6.**
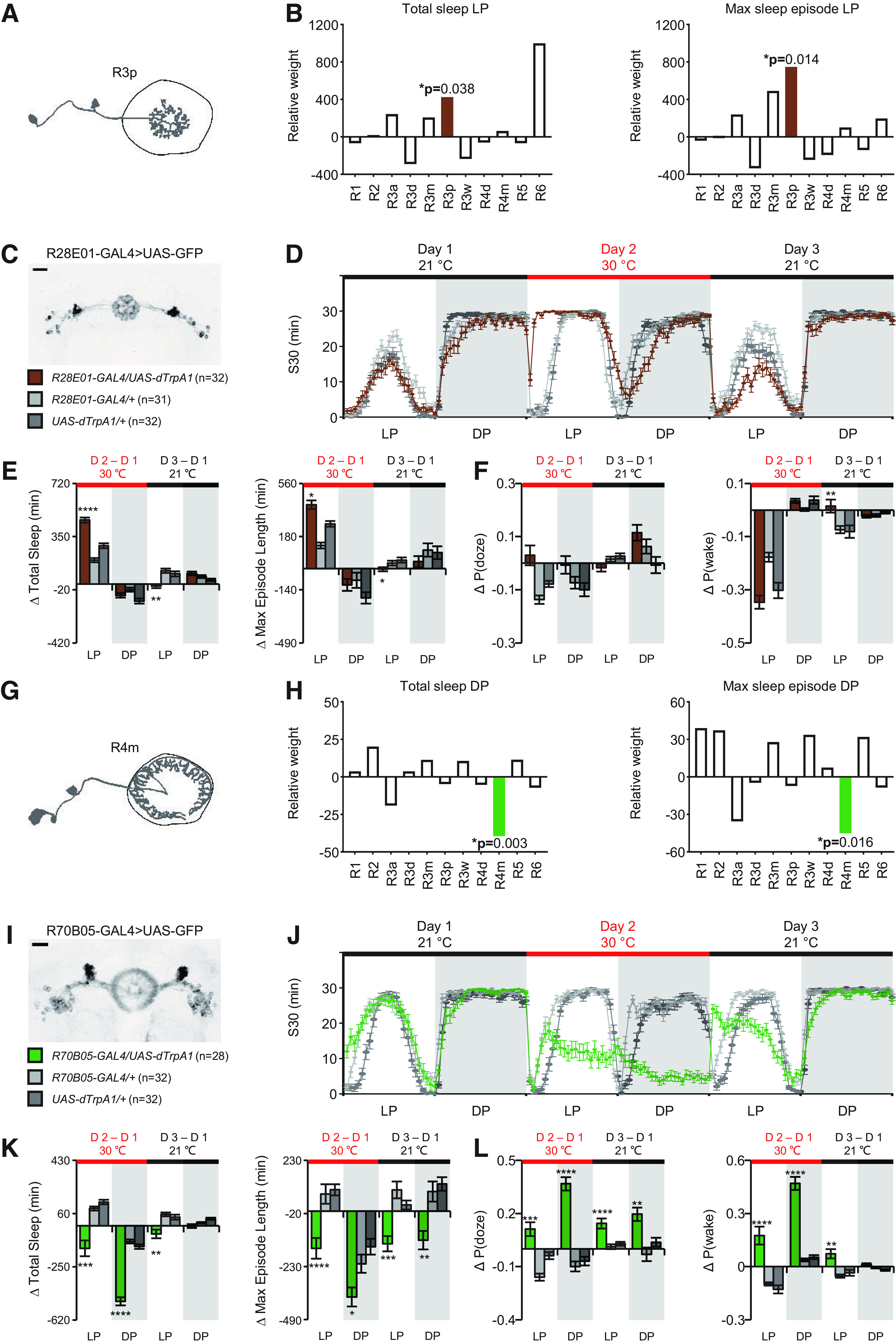
Two subtypes of ring neurons identified by GLM that significantly contribute in the regulation of total sleep and episode length. ***A***, ***G***, Schematic morphologic pattern of a single R3p neuron and R4m neuron, respectively. ***B***, ***H***, R3p neuron and R4m neuron are highly correlated to regulate daytime sleep and nighttime sleep, respectively. The weight of each subclass was analyzed with a GLM. ***C***, ***I***, Expression pattern of R28E01-GAL4 and R70B05-GAL4 as representative for R3p and R4m. ***D***, ***J***, Sleep profiles of total sleep before, during and after activation of R28E01-GAL4^+^ and R70B05-GAL4^+^ neurons with two controls. ***E***, ***K***, Changes in total amount of sleep and maximum episodes on the activation day and the recovery day. R70B05-GAL4^+^ neurons not only significantly reduced nighttime sleep but also exhibited strong impact on reducing daytime sleep. ***F***, No detectable changes in P(doze) on and after activation of R28E01-GAL4^+^ neurons. Weak elevation of P(wake) on the recovery day was found. ***L***, Strong increase in P(doze) was found when R70B05-GAL4^+^ neurons were activated, and this effect lasted with cessation of activation. Significant increase in P(wake) was also observed on and after activation. *p < 0.05. **p < 0.01. ***p < 0.001. ****p < 0.0001. Data are mean ± SEM. Scale bar: 20 μm.

**Figure 7.**
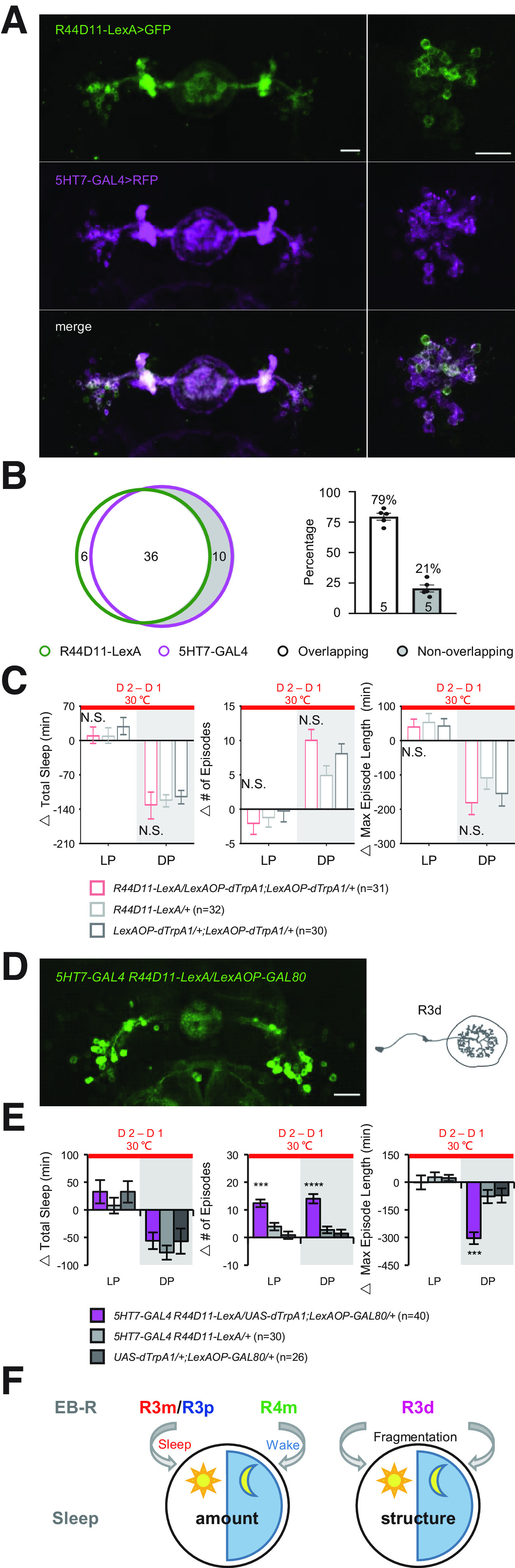
R3d neurons contribute to sleep fragmentation. ***A***, Expression pattern of R44D11-LexA^+^ neurons and 5HT7-GAL4^+^ neurons labeled by GFP and RFP, respectively; 79% of the R44D11-LexA^+^ neurons (green) overlap with 5HT7-GAL4^+^ neurons (magenta). ***B***, Venn diagram represents the overlapping and nonoverlapping cells between 5HT7-GAL4^+^ and R44D11-LexA^+^. Bar graph represents the quantified ratio of nonoverlapping neurons. ***C***, Activation of R44D11-LexA^+^ neurons do not change sleep or sleep structure during both day and night. ***D***, R3d populations are labeled by suppressing the overlapped neurons of R44D11-LexA^+^ and 5HT7-GAL4^+^ by using LexAOP-GAL80. Scale bar, 20 μm. ***E***, Activation of the nonoverlapping R3d neurons fragments sleep without significant effect on total amount during LP and DP. ***F***, Schematic of sleep/structure regulation by multiple subtypes of ring neurons. ***p < 0.001. ****p < 0.0001. Data are mean ± SEM.

The first approach we used was aimed at determining the effects of the GAL4 lines (each of which has a different mixture of ring neuron subtypes) in regulating sleep. We used a mixed Gaussian model for changes in P(wake) or P(doze) on the activation day compared with the baseline day (Fig. 5*A*,*B*). We chose to use these transition probabilities since they capture some of the more complex aspects of sleep: P(wake) correlates with arousal state/sleep depth, while P(doze) is a measure of sleep drive (Wiggin et al., 2020). A single value of ΔP(wake) and ΔP(doze) for each line was calculated by subtracting the average of the genetic controls for that driver (experimental ΔP – (UAS ΔP + GAL4 ΔP)/2)). These values were then plotted in ΔP(wake)–ΔP(doze) space and clustered with the model to find groups with similar effects on sleep depth and pressure. We identified five clusters of GAL4 lines for day and night, respectively (Fig. 5*A*,*B*). These clusters define the color codes used in Figures 1, 2, 3, 5, 6 and Figure 7.

Using our anatomic analysis of these lines, we found that the lines within each cluster shared a common ring neuron subtype. During the daytime (Fig. 5*A*), R4m (and perhaps R2 neurons) emerged as strong candidates for the regulation of sleep depth/arousal since they are present in lines that have high ΔP(wake) values. R3dm cells appeared to increase sleep drive (i.e., increase the probability of falling asleep); consistent with this, lines with these cells had high P(doze). R2, R3d, and R3p neurons were present in several clusters and did not appear to have unique functionality with regard to sleep depth and drive, but a role in facilitation of the effects of R2 and R3m neurons, or in more specialized functions in sleep structure, cannot be ruled out. We also observed that many drivers play different roles during the day and night (Fig. 5*B*). For example, R70B05 exhibits relative strong P(wake) but weak P(doze) effect during the day, but at night increases its influence on P(doze); R47F07 has little effect on P(wake) in the day but becomes much more wake-promoting at night.

### Association of specific ring neuron subtypes with changes in sleep parameters

Since the variable analyzed using Gaussian clustering was the GAL4 line, which is most often a collection of different ring neuron subtypes, the effects we saw could also be the result of particular combinations of subtypes rather than the result of one dominant subtype alone. To try to isolate effects specific to subtypes, and to look at more specific sleep parameters, we used a second method to extract the contributions of each ring neuron subtype to functional outcomes. Using a GLM with ring neuron subtype as the variable allowed us to calculate the weights of the potential contribution of each subtype of ring neuron to all the sleep parameters for daytime and nighttime, respectively (Table 6). R3p exhibited a significantly positive effect on daytime sleep amount which was associated with its positive weight in episode length (Fig. 6*A*,*B*; Table 7). As an example, activation of R28E01-GAL4^+^ neurons, which include the R3p subtype, elevated daytime sleep and maximum episode length (Fig. 6*C–E*; Table 8). But the R3p subtype had little effect on P(doze) or P(wake) (Fig. 6*F*; Table 8). We also found that R4m had a significantly inhibiting effect on total sleep at night and a negative effect on episode length (Fig. 6*G*,*H*; Table 7), consistent with the results of Gaussian clustering. R70B05-GAL4^+^ neurons include the R4m subtype, and activation of neurons labeled by this driver caused a dramatic reduction of sleep in both day and night, which is likely because of the shortened episode length (Fig. 6*I–K*; Table 8). The effects of activation of these R70B05-GAL4^+^ neurons persisted into the recovery day, with flies exhibiting significantly elevated sleep pressure and lightened sleep depth (Fig. 6*L*; Table 8).

**Table 6.** Statistical table of the weight of the effect of subclasses of ring neurons on the sleep using a GLM*^a^*

	Total sleep				No. of episodes				Maximum episode length			
	LP		DP		LP		DP		LP		DP	
Subtype	Weight	*p*	Weight	*p*	Weight	*p*	Weight	*p*	Weight	*p*	Weight	*p*
R1	−66.963	0.806	3.71	0.789	−78.081	0.218	−289.982	0.025*	−40.711	0.918	39.093	0.058
R2	−4.43	0.986	20.206	0.117	−53.171	0.351	−287.956	0.015*	10.987	0.976	37.288	0.047*
R3a	243.881	0.394	−19.084	0.192	0.156	0.998	100.72	0.43	239.529	0.562	−35.538	0.095
R3d	−290.368	0.202	3.727	0.742	72.222	0.166	−99.637	0.323	−335.803	0.306	−4.78	0.769
R3m	207.085	0.45	11.389	0.413	−110.33	0.086	97.25	0.428	490.865	0.222	27.874	0.168
R3p	424.718	0.038*	−4.8	0.628	12.879	0.772	−24.945	0.775	747.409	0.014*	−7.29	0.608
R3w	−235.362	0.422	10.664	0.472	−77.07	0.253	−118.224	0.367	−244.843	0.563	33.605	0.122
R4d	−60.809	0.768	−5.332	0.61	−23.994	0.611	−104.199	0.264	−192.1	0.522	7.413	0.621
R4m	63.512	0.793	−39.476	0.003*	−3.416	0.951	97.465	0.371	100.607	0.774	−45.005	0.016*
R5	−67.36	0.751	11.467	0.292	−49.769	0.31	−168.218	0.086	−140.881	0.648	31.922	0.048*
R6	999.282	0.069	−7.214	0.788	81.007	0.505	103.387	0.663	196.977	0.798	−8.583	0.823

*^a^*The generalized linear model (GLM) analysis was conducted using the script of glmfit in MATLAB with the default parameters setting for total sleep, number of episodes, and maximum episode length. A positive value represents a positive relationship, and a negative value represents a negative relationship between the subtype of ring neurons and the sleep parameter, respectively.

*p < 0.05.

**Table 7.** Power analysis for the sample size of two drivers used in Figure 6*^a^*

Total sleep	Experiment vs GAL4 Control				Experiment vs UAS Control			
	30°C		21°C		30°C		21°C	
Drivers	LP	DP	LP	DP	LP	DP	LP	DP
R28E01	1	0.511	0.999	0.28	1	0.313	0.9	0.951
R70B05	1	1	1	0.667	1	1	0.994	0.985

No. of episodes	Experiment vs GAL4 Control				Experiment vs UAS Control			
	30°C		21°C		30°C		21°C	
Drivers	LP	DP	LP	DP	LP	DP	LP	DP
R28E01	0.116	0.101	0.198	0.233	0.425	0.074	0.214	0.121
R70B05	0.992	0.888	1	0.994	0.47	0.878	1	0.997

Maximum episode length	Experiment vs GAL4 Control				Experiment vs UAS Control			
	30°C		21°C		30°C		21°C	
Drivers	LP	DP	LP	DP	LP	DP	LP	DP
R28E01	1	0.07	0.425	0.222	0.995	0.354	0.659	0.161
R70B05	0.947	0.652	0.981	0.873	1	0.983	1	1

*^a^*Two GAL4 drivers were included in the analysis: R28E01 and R70B05. The experimental group was compared with either GAL4 control group or UAS-dTrpA1 control group for both activation day and recovery day. Total sleep, number of episodes, and maximum episode length for LP and DP were analyzed separately. Power analysis was conducted using the script of sampsizepwr in MATLAB.

**Table 8.** Statistical analysis of the recovery day for two drivers used in Figure 6*^a^*

	LP										DP					
Driver	Nonparametric/parametric test				*Post hoc* comparisons						Nonparametric/parametric test			*Post hoc* comparisons		
D3-D1 21°C	Test	DFn, DFd	*F*	*p*		n1	n2	Mean difference	*p*		Test	*F*	*p*	Mean difference	*p*	
R28E01																
△ Total sleep	K-W	3,95	22	<0.0001	1 vs 2	32	31	−31.06	<0.0001	****	K-W	4.966	0.0835	5.503	0.8565	NS
					1 vs 3	32	32	−23.77	0.0011	**				15.17	0.0554	NS
△ Maximum episode length	K-W	3,95	7.5	0.0235	1 vs 2	32	31	−15.71	0.0474	*	K-W	1.406	0.4951	−7.219	0.5974	NS
					1 vs 3	32	32	−16.97	0.0276	*				−6.969	0.6239	NS
△ P(doze)	K-W	3,96	4.376	0.1121	1 vs 2	32	32	−7.656	0.5432	NS	K-W	8	0.0183	9.281	0.3653	NS
					1 vs 3	32	32	−14.56	0.073	NS				19.69	0.0094	**
△ P(wake)	K-W	3,96	12.76	0.0017	1 vs 2	32	32	22.16	0.0029	**	K-W	2.335	0.3111	−2.031	>0.9999	NS
					1 vs 3	32	32	20.88	0.0054	**				−10.06	0.297	NS
R70B05																
△ Total sleep	K-W	3,92	16.31	0.0003	1 vs 2	28	32	−26.52	0.0002	***	K-W	13.3	0.0013	−7.692	0.5311	NS
					1 vs 3	28	32	−21.48	0.0038	**				−24.4	0.0008	***
△ Maximum episode length	K-W	3,92	27.48	<0.0001	1 vs 2	28	32	−35.09	<0.0001	****	K-W	20.64	<0.0001	−23.01	0.0017	**
					1 vs 3	28	32	−26.31	0.0003	***				−30.33	<0.0001	****
△ P(doze)	ANOVA	2,86	14.35	<0.0001	1 vs 2	27	30	0.1319	<0.0001	****	ANOVA	10.82	<0.0001	0.2261	<0.0001	****
					1 vs 3	27	32	0.1144	<0.0001	****				0.1582	0.0035	**
△ P(wake)	K-W	3,89	18.94	<0.0001	1 vs 2	27	30	28.97	<0.0001	****	K-W	14.74	0.0006	15.24	0.0523	NS
					1 vs 3	27	32	21.15	0.0035	**				25.88	0.0003	***

*^a^*Change in sleep parameters, total sleep, number of episodes, maximum episode length, P(doze), and P(wake) on the recovery day (21°C) were analyzed for day (LP) and night (DP) separately. One-way ANOVA followed by Bonferroni test or Kruskal–Wallis (K-W) followed by Dunn's test was applied based on distribution of the datasets.

*p < 0.05.

**p < 0.01.

***p < 0.001.

****p < 0.0001.

### Ring neuron synergy is important for sculpting sleep

Interestingly, there were effects uncovered in the GLM analysis that were not seen with GAL4 drivers that labeled only that specific subtype. R1 and R2 neurons exhibit a significantly negative weight in the number of episodes at night, suggesting that these neurons may contribute to consolidation of sleep structure (Table 6). However, we failed to observe consolidation after activation of R1- or R2-specific GAL4 drivers; R56C09 and R81F01 had little significant effect on sleep structure (Fig. 1; Table 1), while activation of R48B10 produced a moderately strong increase in P(doze/wake) (Fig. 2*M*; Table 3). This suggests that the sleep consolidation effects of activating these neurons uncovered by the GLM requires coactivation of other subtypes.

Supporting the complexity of ring neuron subtype interactions, we observed that activation of the R47F07-GAL4 driver, which labels R3a, R3m, and R3p ring neurons, induced increased daytime sleep but reduced nighttime sleep (Figs. 3*E*,*F*, 5; Table 4). Increased daytime sleep was associated with an increase of episode length, explained by elevated sleep pressure and “deeper” sleep depth (Figs. 3*G*,*H*, 5; Table 4). Opposite to the daytime change, reduced nighttime sleep was accompanied by fragmentation, resulting in increased sleep pressure and/or light sleep depth (Figs. 3*G*,*H*, 5; Table 4). How these three subtypes of ring neurons coordinate to segregate, and effect a sign change on, day and night sleep still needs to be determined but may provide insight into coordination of the EB circuit.

### Regulation of sleep fragmentation by a specific ring neuron subset

One of the interesting findings of this screen was that there appeared to be circuits that regulate sleep structure independent of sleep amount. These data were consistent with our previous studies, which identified 5HT in EB as a modulator of sleep structure; activation of 5HT7-GAL4^+^ neurons fragmented sleep without changing the amount of sleep (C. Liu et al., 2019). 5HT7-GAL4^+^ neurons include R3d, R3p, and R4d subtypes (Hulse et al., 2021). To examine whether sleep structure regulation could be attributed to a specific subtype, we identified a driver R44D11-LexA that had an expression pattern similar to 5HT7-GAL4 (Fig. 7*A*). LexA^+^ neurons overlapped nearly 79% with 5HT7-GAL4^+^ neurons (Fig. 7*B*), but activation of R44D11-LexA^+^ neurons does not induce sleep/structure changes on activation (Fig. 7*C*; Table 9). To test the hypothesis that sleep fragmentation might be induced by the nonoverlapping population of 5HT7-GAL4^+^ neurons, we introduced LexAop-GAL80 to suppress the overlapping neurons between R44D11-LexA^+^ and 5HT7-GAL4^+^ neurons (Fig. 7*D*). We found that activation of the nonoverlapping 5HT7-GAL4^+^ neurons increased the number of episodes and reduced episode length (Fig. 7*E*; Table 9), suggesting that the nonoverlapping neurons play a critical role in sleep fragmentation. Interestingly, the nonoverlapping neurons morphologically are R3d subtypes (Fig. 7*D*). This subtype of ring neuron was present in 4 of 6 of the lines we identified in this screen as affecting structure only (R70B04, R53F11, R54B05, R53G11), and there were also R3d neurons in some lines that fragmented sleep in addition to changing its amount (Aphc507, R84H09). The fact that not all lines that contain this ring neuron subtype fragment sleep may be because of interactions with other ring neuron types or heterogeneity within the R3d population.

**Table 9.** Statistical analysis of the activation day for two drivers used in Figure 7*^a^*

	LP										DP					
	Nonparametric/parametric test				*Post hoc* comparisons						Nonparametric/parametric test			*Post hoc* comparisons		
D3-D1 21 °C	Test	DFn, DFd	*F*	*p*		n1	n2	Mean difference	*p*		Test	*F*	*p*	Mean difference	*p*	
Figure 7C																
△ Total sleep	K-W	3,93	2.44	0.2952	1 vs 2	31	32	0.2535	>0.9999	NS	K-W	4.394	0.1111	−10.63	0.2363	NS
					1 vs 3	31	30	−9.22	0.3644	NS				−13.76	0.0929	NS
△ No. of episodes	K-W	3,93	0.7446	0.6892	1 vs 2	31	32	−1.765	>0.9999	NS	K-W	7.075	0.0291	17.92	0.0167	*
					1 vs 3	31	30	−5.817	0.799	NS				7.035	0.6165	NS
△ Maximum episode length	K-W	3,93	0.1451	0.93	1 vs 2	31	32	−2.587	>0.9999	NS	ANOVA	1.287	0.281	−72.87	0.202	NS
					1 vs 3	31	30	−1.191	>0.9999	NS				−26.9	0.7888	NS
Figure 7E																
△ Total sleep	ANOVA	2,93	0.5201	0.5962	1 vs 2	40	30	25.21	0.5593	NS	K-W	1.289	0.525	7.621	0.5146	NS
					1 vs 3	40	26	0.2596	>0.9999	NS				2.791	>0.9999	NS
△ No. of episodes	K-W	3,96	27.92	<0.0001	1 vs 2	40	30	24.85	0.0004	***	ANOVA	21.09	<0.0001	11.26	<0.0001	****
					1 vs 3	40	26	34.78	<0.0001	****				12.6	<0.0001	****
△ Maximum episode length	K-W	3,96	1.574	0.4551	1 vs 2	40	30	−5.592	0.8118	NS	K-W	20.92	<0.0001	−25.46	0.0003	***
					1 vs 3	40	26	−8.41	0.4615	NS				−27.34	0.0002	***

*^a^*Change in sleep parameters, total sleep, number of episodes, and maximum episode length on the activation day (30°C) were analyzed for LP and DP separately. One-way ANOVA followed by Bonferroni test or Kruskal–Wallis (K-W) followed by Dunn's test was applied based on distribution of the datasets.

*p < 0.05.

***p < 0.001.

****p < 0.0001.

## Discussion

Sleep is crucial for survival and overall health across animal kingdoms. Fly sleep exhibits the majority of the highly conserved features of vertebrate sleep, and the tractability of *Drosophila* as an experimental model has produced a growing number of studies, which contribute to our knowledge of sleep mechanisms and circuits. In addition to the importance in learning and memory of the mushroom body (MB), multiple subtypes of intrinsic MB Kenyon cells (KCs) have been identified as influencing sleep (Joiner et al., 2006; Sitaraman et al., 2015; Artiushin and Sehgal, 2017; Bringmann, 2018). For example, α′β′ and γm KCs contribute to wake promotion, and γd KCs contribute to sleep promotion (Sitaraman et al., 2015). A pair of GABAergic and serotonergic dorsal paired medial neurons, which are MB extrinsic projecting neurons and play a role in memory consolidation (Keene et al., 2004, 2006; Zhang et al., 2013), were shown to be involved in promoting sleep (Haynes et al., 2015). Dopaminergic PPL1 and PPM3 neurons that project to different layers of fan-shaped body (FB) have been shown to have specific roles in wake, via suppression of the FB, which is thought as a sleep-induction center (Q. Liu et al., 2012; Ueno et al., 2012; Pimentel et al., 2016). In addition to these central neurons, peripheral neurons, such as ppk^+^ neurons that project to the central brain, have been shown to have a role in the regulation of sleep homeostasis (Satterfield et al., 2022).

Many of these brain structures have been implicated in multiple behaviors. Like the MB and FB mentioned above, the EB has been shown to integrate sensory inputs to formulate locomotor output commands, but our understanding of its role in sleep is still limited. In the present study, we identified subtypes of ring neurons that regulate sleep/structure by the following: (1) screening a small collection of EB drivers using thermogenetic activation; and (2) specifying the roles of several single subtypes in different sleep components using two models and intersection strategies. We found that R3m/R3p neurons contribute to daytime sleep, R4m neurons to wakefulness, and R3d neurons fragment sleep structure (Fig. 7*F*).

The role of these neurons in sleep may be intimately involved with their other functions. Previous studies found that R2, R3, R4d, and R4m subtypes appear to be tuned to visual stimuli (Shiozaki and Kazama, 2017; Fisher et al., 2019; Kim et al., 2019; Hardcastle et al., 2021). This sensory input may be an important cue to change sleep/wake status, and is likely influenced by the circadian system. Previous studies showed that the R5 subtype is linked to the control of sleep homeostasis and stabilization of sleep structure (S. Liu et al., 2016; C. Liu et al., 2019), and our analysis supports these findings. A recent study released on *bioRxiv* identified two subtypes: sleep-promoting R3m neurons and wake-promoting R3d neurons (Aleman et al., 2021). Consistently, we also observed that R3m contributes both sleep amount and sleep structure. 5HT7-GAL4^+^ neurons play an important role in sleep maintenance, when they are activated, sleep became fragmented (C. Liu et al., 2019). According to a recent anatomic analysis (Hulse et al., 2021), 5HT7-GAL4^+^ neurons include R3d, R3p, and R4d subtypes, and we narrowed the fragmentation effect down to a specific subtype (R3d) in the present study. However, more efforts are still needed to understand how a certain subtype of ring neuron responds to sensory inputs and how neuronal activity patterns form in the network. Future work examining the neural activity of each subtype of ring neurons that control distinct sleep components and the interaction with other behaviors may reveal fundamental information about the rules of the coding and integration of the brain.
